# A systematic review and network meta-analysis of current and investigational treatments for active ankylosing spondylitis

**DOI:** 10.1007/s10067-020-04970-3

**Published:** 2020-02-27

**Authors:** A. Deodhar, S. D. Chakravarty, C. Cameron, S. Peterson, R. Hensman, S. Fogarty, P. Spin, S. Kafka, S. Nair, L. S. Gensler

**Affiliations:** 1 Oregon Health & Science University, Portland, OR USA; 2 Janssen Scientific Affairs, LLC, Horsham, PA USA; 3 Drexel University College of Medicine, Philadelphia, PA USA; 4 Cornerstone Research Group Inc., Burlington, Ontario Canada; 5 Janssen Global Services, LLC, Horsham, PA USA; 6 Janssen Pharmaceutica NV, Turnhoutseweg 30, 2340 Beerse, Belgium; 7 University of California San Francisco, San Francisco, CA USA

**Keywords:** Ankylosing spondylitis, Biologic, Intravenous golimumab, Network meta-analysis, Systematic review

## Abstract

**Objective:**

To compare the relative efficacy of current and investigational biologic and oral small molecule (OSM) treatments for active ankylosing spondylitis (AS).

**Methods:**

A systematic literature review was conducted to identify all phase 2/3 randomized trials of interest in patients with AS. Outcomes assessed were ≥ 20% improvement in the Assessment of Spondyloarthritis International Society Criteria (ASAS20) and change from baseline in Bath Ankylosing Spondylitis Functional Index (BASFI) and C-reactive protein (CRP) at weeks 12–16. Bayesian network meta-analyses were conducted for outcomes using a random effects model. Baseline-risk adjustment was also conducted to account for differences in placebo response across studies. Surface Under the Cumulative Ranking curve (SUCRA) values are reported, reflecting the relative probability that intervention was the best of all interventions.

**Results:**

The investigational agent tofacitinib 5 mg was the top-ranked treatment (SUCRA, 93%) for ASAS20 response, followed by intravenous (IV) golimumab 2 mg/kg (90%). Golimumab IV 2 mg/kg and infliximab 5 mg/kg were the top two ranked treatments for change from baseline in BASFI (golimumab IV, 81%; infliximab, 80%) and change from baseline in CRP (infliximab, 90%; golimumab IV, 82%).

**Conclusions:**

Two approved therapies (golimumab IV, infliximab) and one investigational product ranked highest for efficacy in AS.

**Table:** 

**Key Points** *• Although golimumab IV, infliximab, and tofacitinib ranked highest for efficacy in AS, differences in efficacy between approved and investigational therapies were not statistically significant.*

**Electronic supplementary material:**

The online version of this article (10.1007/s10067-020-04970-3) contains supplementary material, which is available to authorized users.

## Introduction

Ankylosing spondylitis is a chronic, immune-mediated, inflammatory condition within the family of spondyloarthritis (SpA) with several shared clinical, genetic, and immunologic features [1]. Active ankylosing spondylitis is characterized by inflammation at the sacroiliac joint and insertion sites of tendons and ligaments (i.e., enthesitis), as well as increased risk of fusion of the sacroiliac joint and spine [2, 3]. Patients experience severe back pain, spinal stiffness, and reduced spinal mobility, which may lead to severe deformity (i.e., bamboo spine) in some.

Current guidelines recommend non-steroidal anti-inflammatory drugs (NSAIDs) and physical therapy as first-line treatment; however, some patients still experience active disease [1]. For these patients, biologic therapies targeting two cytokine pathways are available, including five tumor necrosis factor (TNF) inhibitors [4–9] and one interleukin (IL)-17 inhibitor [10]. Janus kinase (JAK) inhibitors [11, 12], two IL-17 inhibitors [13, 14], and one IL-23 inhibitor [15] are currently under investigation for the treatment of AS.

Because of the lack of head-to-head studies that directly compare many of the available biologic therapies and oral small molecules (OSM) for active ankylosing spondylitis, systematic literature reviews (SLRs) and Bayesian network meta-analyses (NMAs) have been conducted to evaluate relative efficacy. Recent SLRs and NMAs have compared efficacy within a class of therapies (i.e., TNF inhibitors) and across all then-available biologics for the treatment of ankylosing spondylitis [16, 17]. Since these publications, the intravenous (IV) formulation of the TNF inhibitor golimumab (GOL IV) was approved by the US Food and Drug Administration (FDA) for the treatment of adults with active ankylosing spondylitis. Additionally, data from recently published phase 3 clinical trials for ixekizumab (IXE) [18, 19] and ustekinumab (UST) [19], as well as phase 2 data for risankizumab (RIS) [15], tofacitinib (TOF) [12], and filgotinib (FIL) [11], have become available.

The objective of this study was to conduct a comprehensive comparison of all current and investigational treatments for active ankylosing spondylitis based on all available phase 2/3 data for interventions of interest using a Bayesian NMA.

## Materials and methods

### Systematic literature review

#### Search strategy

An SLR was conducted to identify all phase 2/3 randomized controlled trials (RCTs) that compared the efficacy of biologics and OSMs in the treatment of active ankylosing spondylitis. A strategy was developed by an information specialist and searches were conducted on May 28, 2018, and November 5, 2018, using the OVID platform to search OVID MEDLINE, including Epub Ahead of Print, In-Process and Other Non-Indexed Citations, Embase, and the CENTRAL Database of the Cochrane Library (Wiley version) (Appendix S1). The search strategies used a combination of controlled vocabulary (“Spondylitis, Ankylosing”) and keywords (e.g., “ankylosing spondylitis,” “ankylopoietic spondylarthritis”, “Bechterew”). Vocabulary and syntax were adjusted across databases and results were limited from April 1, 2016, to November 5, 2018. An amended version of the 2008 Cochrane Highly Sensitive Search Strategy (sensitivity, and precision-maximizing version) was applied to the Ovid searches. Where applicable, animal-only records were removed from the results. Randomized controlled trials published before 2016 were identified through review of bibliographies of relevant SLRs and meta-analyses, such as Wang et al. [16] A supplementary search of clinicaltrials.gov was conducted on November 26, 2018, to obtain any additional relevant references.

#### Selection criteria

Eligibility criteria were developed as follows and used to screen all identified studies. The study population was defined as adults (≥ 18 years) diagnosed with active ankylosing spondylitis. Interventions of interest were adalimumab (ADA), apremilast (APR), certolizumab pegol (CZP), etanercept (ETN), filgotinib, infliximab (IFX), ixekizumab, GOL (both IV and subcutaneous (SC) formulations), placebo (PBO), risankizumab, secukinumab (SEC), tofacitinib, and ustekinumab. Outcomes of interest were predefined as an improvement of ≥ 20% in the Assessment of Spondyloarthritis International Society Criteria (ASAS20), change in Bath Ankylosing Spondylitis Functional Index (BASFI), and change in C-reactive protein (CRP) at weeks 12 to 16. All included studies were published full-text RCTs. Although the IL-17A and IL-17F inhibitor bimekizumab is in clinical development, it was not included because a peer-reviewed publication on the use of this agent in AS is not currently available. Conference abstracts were excluded.

#### Study selection

An independent review of identified titles and abstracts was conducted in duplicate to identify studies matching the predefined eligibility criteria. Eligible records were reviewed in full text by the same two reviewers. Conflicts between reviewers were resolved through a consensus meeting.

The study selection process was illustrated in a modified Preferred Reporting Items for Systematic Reviews and Meta-Analyses (PRISMA) flow diagram. Data from eligible articles were collected using a data extraction form. In addition to the outcomes of interest listed above, study design and patient baseline characteristics were extracted to assess the comparability of studies and identify the presence of heterogeneity. Data extraction was performed by a single reviewer and confirmed by a second reviewer. The quality of each study was assessed using the Cochrane Risk of Bias Assessment Tool for Randomized Controlled Trials.

#### Ethics

Ethics was not a requirement given analyses based on publicly available summary-level data.

### Network meta-analysis

NMAs were conducted using Bayesian methods. In a Bayesian NMA, a posterior distribution for each treatment effect is derived by combining a prior probability distribution (i.e., a prior belief of the relative efficacy of a treatment) with a likelihood (i.e., a statistical model) for the effect estimate. A non-informative prior, which assigns an equal probability to any theoretically plausible effect estimate, was used to avoid undue influence of an overly informative prior distribution. Given the data and likelihood, a posterior distribution consisting of 15,000 effect estimates (i.e., mean difference (MD) for continuous endpoints or odds ratio (OR) for binary endpoints) was estimated using Markov Chain Monte Carlo re-sampling methods. This distribution was then used to obtain the median effect estimate (i.e., a point estimate) and the 95% credible interval (CrI), which includes all posterior values between the 2.5th and 97.5th percentiles of the posterior distribution.

Bayesian NMAs were conducted for ASAS20 response, change from baseline in BASFI, and change from baseline in CRP using a random effects (RE) model. ASAS20 response was defined as a ≥ 20% relative improvement from baseline and absolute improvement from baseline of ≥ 10 units on a 0–100mm scale in ≥ 3 of the following domains: patient global assessment, spinal pain assessment, function (BASFI), and inflammation (the last two questions of BASDAI) [20]. Pairwise comparisons between interventions were reported as median OR with 95% CrIs for ASAS20 and as MD with 95% CrIs for change from baseline in BASFI and change from baseline in CRP. The Surface Under the Cumulative Ranking curve (SUCRA) values, reported as percentages, were calculated to reflect the relative probability of an intervention being among the best options. Each NMA was performed in accordance with the methodology recommended by the National Institute for Health and Care Excellence (NICE). To adjust for differences in placebo response across studies, a baseline risk-adjusted sensitivity analysis was conducted using code reported in the NICE Decision Support Unit (DSU) Technical Support Document (TSD) 3. Deviance information criterion (DIC) and posterior residual deviance were reported to assess model fit for each analysis. The estimated regression coefficient (β) and 95% CrIs were reported to assess model fit for baseline risk-adjusted analyses. All analyses were performed using WinBUGS [21] and R [22] with burn-in and sampling durations ≥ 50,000 iterations. Comparisons of baseline risk-adjusted and unadjusted NMAs were used to assess risk of bias from between-study heterogeneity. Inconsistency models were developed and compared to consistency models to assess inconsistency in the networks.

## Results

### Study selection

A total of 1466 published manuscripts were identified from database searches and 104 from supplementary searches. A total of 1570 records were screened at the title and abstract stage and 276 were included for full-text review. Of those reviewed in full text, 244 records were excluded with reason and 32 records, representing 30 unique studies, were included in the final review (Appendix S1).

### Study and patient characteristics

The 30 unique RCTs enrolled a total of 6711 patients. A summary of study and patient baseline characteristics is presented in Appendix S2. The mean age of patients ranged from 27.4 to 48.0 years and the mean disease duration ranged from 5.2 to 23.0 years. The proportion of male patients ranged from 52.6 to 100% and the proportion of HLA-B27-positive patients ranged from 65.0 to 97.4%. Mean baseline scores for Bath Ankylosing Spondylitis Disease Activity Index (BASDAI) ranged from 4.4 to 7.6 cm, for BASFI ranged from 3.2 to 7.4 cm, and for CRP ranged [23] from 6.2 to 32.0 mg/l. Of the 30 studies, 23 allowed concomitant use of conventional synthetic disease-modifying antirheumatic drugs (csDMARDs), four prohibited concomitant use, and three did not report this information.

The risk of bias was generally low across trials for random sequence generation, allocation concealment, incomplete outcomes, and selective reporting (Appendix S4).

### NMA results

**Fig. 1:**
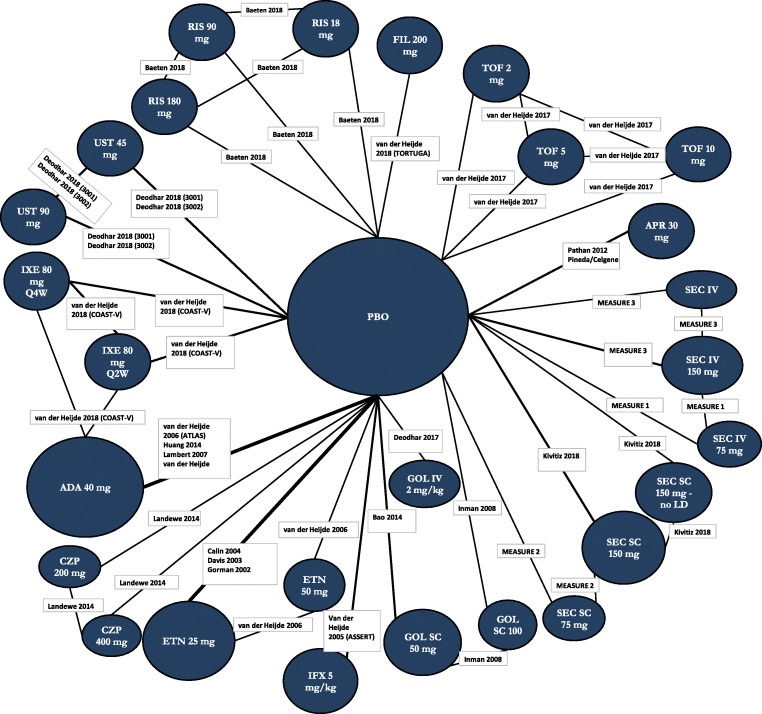
Evidence network for ASAS20 NMA. Each intervention is represented by a node and randomized comparisons are shown as links between the nodes. The size of each node represents the number of patients randomized to each treatment, and the width of connections is reflective of number of RCTs. Abbreviations: ADA, adalimumab; APR, apremilast; ASAS20, improvement of ≥ 20% in the Assessment of Spondyloarthritis International Society Criteria; CZP, certolizumab pegol; ETN, etanercept; FIL, filgotinib; GOL, golimumab; IFX, infliximab; IV, intravenous; IXE, ixekizumab; LD, loading dose; NMA, network meta-analysis; PBO, placebo; Q2W, every 2 weeks; Q4W, every 4 weeks; RIS, risankizumab; TOF, tofacitinib; SC, subcutaneous; SEC, secukinumab; UST, ustekinumab

**Fig. 2:**
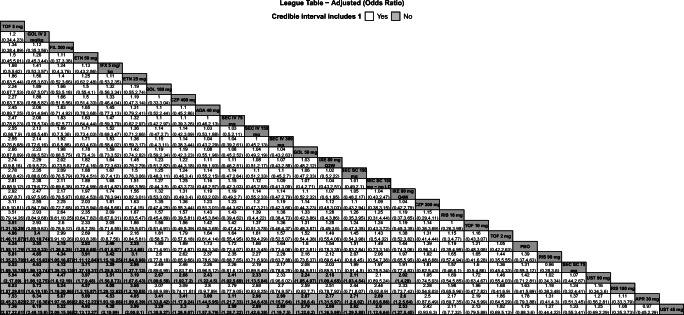
League table of pairwise comparisons for all treatments in the ASAS20 NMA. Pairwise comparisons are reported as ORs and 95% Crls. Comparators are ordered from largest (top-left) to smallest (bottom-right) SUCRA values for the ASAS20 NMA. Please refer to Fig. 5 for SUCRA values for each NMA. Superior improvements in ASAS20 are denoted in bold text and light gray cells. Results are shown for the baseline risk-adjusted model for ASAS20. Please refer to Appendix S6 for the model fit statistics. Abbreviations: ADA, adalimumab; APR, apremilast; ASAS20, improvement of ≥ 20% in the Assessment of Spondyloarthritis International Society Criteria; Crls, credible intervals; CZP, certolizumab pegol; ETN, etanercept; FIL, filgotinib; GOL, golimumab; IFX, infliximab; IXE, ixekizumab; IV, intravenous; LD, loading dose; NMA, network meta-analysis; OR, odds ratio; PBO, placebo; Q2W, every 2 weeks; Q4W, every 4 weeks; RIS, risankizumab; SC, subcutaneous; SEC, secukinumab; SUCRA, Surface Under the Cumulative Ranking curve; TOF, tofacitinib; UST, ustekinumab

**Fig. 3:**
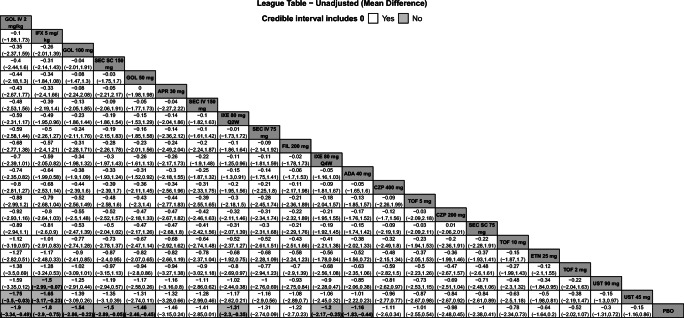
League table with pairwise comparisons for all treatments in the BASFI NMA. Pairwise comparisons are reported as MDs and 95% Crls. Comparators are ordered from largest (top-left) to smallest (bottom-right) SUCRA values for the BASFI NMA. Please refer to Fig. 5 for SUCRA values for each NMA. Superior improvements in BASFI are denoted in bold text and light gray cells. Results are shown for the unadjusted model for BASFI. Please refer to Appendix S6 for the model fit statistics. Abbreviations: ADA, adalimumab; APR, apremilast; BASFI, Bath Ankylosing Spondylitis Functional Index; Crls, credible intervals; CZP, certolizumab pegol; ETN, etanercept; FIL, filgotinib; GOL, golimumab; IV, intravenous; IFX, infliximab; IXE, ixekizumab; MD, mean difference; NMA, network meta-analysis; PBO, placebo; Q2W, every 2 weeks; Q4W, every 4 weeks; SC, subcutaneous; SEC, secukinumab; SUCRA, Surface Under the Cumulative Ranking curve; TOF, tofacitinib; UST, ustekinumab

**Fig. 4:**
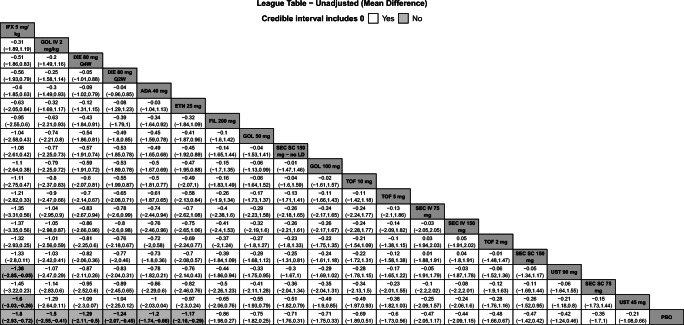
League table with pairwise comparisons for all treatments in the CRP NMA. Pairwise comparisons are reported as MDs and 95% Crls. Comparators are ordered from largest (top-left) to smallest (bottom-right) SUCRA values for the CRP NMA. Please refer to Fig. 5 for SUCRA values for each NMA. Superior improvements in CRP are denoted in bold text and light gray cells. Results are shown for the unadjusted model for CRP. Please refer to Appendix S6 for the model fit statistics. Abbreviations: ADA, adalimumab; CRP, C-reactive protein; Crls, credible intervals; ETN, etanercept; FIL, filgotinib; GOL, golimumab; IFX, infliximab; IV, intravenous; IXE, ixekizumab; LD, loading dose; MD, mean difference; NMA, network meta-analysis; PBO, placebo; Q2W, every 2 weeks; Q4W, every 4 weeks; TOF, tofacitinib; SC, subcutaneous; SEC, secukinumab; SUCRA, Surface Under the Cumulative Ranking curve; UST, ustekinumab

An NMA was conducted for each outcome of interest: attainment of ASAS20 response, change from baseline in BASFI, and change from baseline in CRP. The network diagram for ASAS20 is provided in Fig. 1. Each intervention is represented by a node, and randomized comparisons are shown as links between the nodes. Results of each NMA are presented in Figs. 2, 3, and 4 for the models with the best fit. An odds ratio greater than one or a mean difference less than 0 implies that the reference treatment was better than the comparator. Model fit statistics for each analysis are provided in Appendix S6. A significant β for a baseline risk-adjusted NMA suggested that the adjusted model was a better fit than the unadjusted model.

### ASAS20

**Fig. 5:**
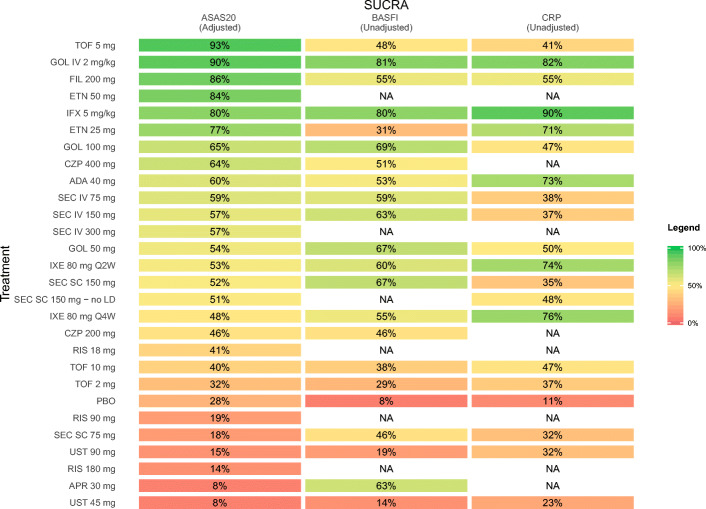
Heat map of SUCRA values for all treatments for each NMA. Results are color coded such that comparators with larger SUCRA values are in green and those with a lower SUCRA values are shown in red. Results of best-fitting model are presented. Results are shown for the baseline risk-adjusted model for ASAS20 and the unadjusted models for change in BASFI and change in CRP. Please refer to Appendix S6 for the model fit statistics. Abbreviations: ADA, adalimumab; APR, apremilast; ASAS20, improvement of ≥ 20% in the Assessment of Spondyloarthritis International Society Criteria; BASFI, Bath Ankylosing Spondylitis Functional Index; CRP, C-reactive protein; CZP, certolizumab pegol; ETN, etanercept; FIL, filgotinib; GOL, golimumab; IFX, infliximab; IV, intravenous; IXE, ixekizumab; LD, loading dose; NA, not available; NMA, network meta-analysis; PBO, placebo; Q2W, every 2 weeks; Q4W, every 4 weeks; RIS, risankizumab; SC, subcutaneous; SEC, secukinumab; SUCRA, Surface Under the Cumulative Ranking curve; TOF, tofacitinib; UST, ustekinumab

Twenty-six studies were included in the comparison of ASAS20 at weeks 12 to 16 (Fig. 1). The baseline risk-adjusted model had the best fit because the 95% CrI for β excluded 0 (Appendix S6). Tofacitinib 5 mg had superior ASAS20 response compared to GOL IV 2 mg/kg and TOF 10 mg, and both TOF 5 mg and GOL IV 2 mg/kg were superior to TOF 2 mg, PBO, RIS 90 mg, SEC SC 75 mg, UST 90 mg, RIS 180 mg, APR 30 mg, and UST 45 mg (Fig. 2**)**. Tofacitinib 5 mg and GOL IV 2 mg/kg were of greater or equal efficacy compared to the other treatments. Rankings based on SUCRA values for ASAS20 response were highest for TOF 5 mg (93%), GOL IV 2 mg/kg (90%), and FIL 200 mg (86%) (Fig. 5).

### Change from baseline in BASFI

Twenty-three studies were included in the comparison of the change from baseline in BASFI at weeks 12 to 16 (Appendix S5A). The unadjusted model had the best fit because the 95% CrI for β excluded 0 (Appendix S6). Golimumab IV 2 mg/kg was ranked highest among all treatments, followed by IFX 5 mg/kg. Both interventions had superior reductions from baseline for BASFI compared to PBO and UST 45 mg, and IFX 5 mg/kg was also superior to UST 90 mg (Fig. 3). SUCRA values for the change from baseline in BASFI were highest for GOL IV 2 mg/kg (81%), IFX 5 mg/kg (80%), and GOL SC 100 mg (69%) (Fig. 5).

### Change from baseline in CRP

Nineteen studies were included in the comparison of the change from baseline in CRP at weeks 12 to 16 (Appendix S5B). The unadjusted model had the best fit because the 95% CrI for β excluded 0 (Appendix S6). Infliximab 5 mg/kg was ranked highest among all treatments, followed by GOL IV 2 mg/kg. Infliximab 5 mg/kg showed superior reduction from baseline in CRP compared to UST 90 mg, UST 45 mg, and PBO and GOL IV 2 mg/kg was superior to PBO (Fig. 4). SUCRA values for the change in CRP NMA were highest for IFX 5 mg/kg (90%), GOL IV 2 mg/kg (82%), and IXE 80 mg Q4W (76%) (Fig. 5).

### Additional analyses

Between-study heterogeneity was assessed for each outcome using the *I*^2^ statistic for pairwise comparisons to placebo with ≥ 2 independent studies (Appendix S10) [23]. The *I*^2^ statistic was low (< 40%) or moderate (30 to 60%) for most comparisons, suggesting that heterogeneity was unlikely to bias the results. Substantial heterogeneity (*I*^2^ > 60%) was observed only in pairwise comparisons of treatments with low SUCRA values which suggests heterogeneity is unlikely to alter conclusions. A comparison of unadjusted and baseline risk-adjusted analyses for each outcome showed that results were similar between models overall (Appendices S6 and S7). For ASAS20, the baseline risk-adjusted NMA yielded smaller heterogeneity compared to the unadjusted NMA. For change in BASFI and CRP, the unadjusted and baseline risk-adjusted models yielded similar results. A sensitivity analysis including studies reporting results at 10 weeks was conducted for each outcome. This sensitivity was conducted to align with the most recently published NMA, Wang et al. [16], which included studies reporting results between 10 to 16 weeks. Inclusion of studies reporting results at 10 weeks resulted in the addition of one new study (Marzo-Ortega 2005 [24]) in the ASAS20, change from baseline in BASFI, and change from baseline in CRP networks. Results of these analyses were similar to those from the reference analyses on all three outcomes (Appendix S7).

## Discussion

This systematic review and NMA compared the efficacy of all current and investigational treatment options for ankylosing spondylitis by assessing ASAS20 response, change from baseline in BASFI, and change from baseline in CRP. Results of the best-fitting model for each network showed that TOF 5 mg and GOL IV 2 mg/kg were the two top-ranked treatments for ASAS20 response, GOL IV 2 mg/kg and IFX 5 mg/kg were the two top-ranked treatments for change from baseline in BASFI, and IFX 5 mg/kg and GOL IV 2 mg/kg were the two top-ranked treatment for change from baseline in CRP.

Tofacitinib 5 mg was ranked first in the baseline risk-adjusted model for ASAS20; however, this was likely due to the higher placebo response rate observed in the TOF study, which shifted the results in favor of TOF 5 mg after the adjustment. Further, the only study for TOF was a phase 2 trial [12] with a relatively small patient population, whereas data for other treatments were based on phase 3 studies with larger populations. Thus, favorable results for TOF 5 mg for ASAS20 should be interpreted with caution. Golimumab IV 2 mg/kg was associated with a greater reduction in BASFI than PBO and UST 45 mg and a greater reduction in CRP from baseline than PBO. GOL IV 2 mg/kg had comparable or superior efficacy than all other comparators. Results of the baseline risk-adjusted NMA were consistent with the unadjusted results.

A recent NMA comparing treatments for ankylosing spondylitis published by Wang et al. [16] compared the efficacy of six TNF inhibitors (ADA, CZP, ETN, GOL SC, IFX, and IFX biosimilar) using BASDAI, BASFI, and CRP outcome measures [16]. Results of the 12-week analyses found that IFX had a superior reduction in BASFI from baseline compared to CZP, whereas no difference was found for other treatment comparisons. This reduction may be because approximately half of the patients who received CZP in the included phase 3 study had non-radiographic axial spondyloarthritis with low functional impairment [8]. No difference in the reduction in CRP from baseline was found among compared treatments. These results align with those presented in the current analyses, where no difference in change from baseline in BASFI or CRP between TNF inhibitor treatments was found. It is important to note that the results from Wang et al. are not directly comparable to the analyses presented. First, multiple treatment doses were combined for each intervention in the Wang et al. analyses, whereas each intervention dose was evaluated separately in the present study. Second, the Wang et al. analysis did not restrict studies to phase 2/3 trials and, therefore, included IFX biosimilars in their analyses. In contrast, data informing the analyses presented here were restricted to phase 2/3 trials and excluded biosimilars. The third major difference from the Wang analysis is that our investigation was not restricted to TNF inhibitors, and thus compared a broader scope of treatments.

### Strengths and limitations

To the best of our knowledge, this study is the most recent and comprehensive SLR and NMA comparing both currently available and investigational biologic and OSM treatments for active ankylosing spondylitis. Prior NMAs published in this therapeutic area have been restricted to TNF inhibitors. This study uniquely provides data on the comparative efficacy of treatment options with differing mechanisms of action to better inform treatment decisions. Given the recent advances in research for the treatment of ankylosing spondylitis, this NMA also provides data on the comparative efficacy of newly studied treatments such as GOL IV, FIL, IXE, RIS, and UST. The analyses conducted aligned with best practices for NMAs [25, 26] and used publicly available code to ensure transparency and reproducibility. Furthermore, an adjusted analysis accounting for baseline risk differences between trials was also conducted to ensure baseline differences were not confounding the results. The analysis reported here focuses on published summary-level data from RCTs. There are indirect treatment comparison methodologies, such as matching-adjusted indirect comparisons (MAIC) and propensity score matching (PSM), available that can leverage individual participant data (IPD) from RCTs to adjust for potential heterogeneity between studies. Although MAICs and PSMs can better adjust for heterogeneity than NMA by leveraging IPD, there was limited heterogeneity identified in the current analysis. Further, MAICs and PSMs only permit comparison of one treatment at a time. In contrast, NMA provides a more holistic view of the body of evidence in ankylosing spondylitis. This is the first NMA to simultaneously compare current and investigational treatments.

There were limited clinical trial data for each treatment to inform the comparative efficacy of biologic and OSM therapies for active ankylosing spondylitis. This is evident from the presence of many single study connections in each network, which limits the strength of the analysis. Insufficient clinical data also limited the choice of outcomes presented in this analysis. All three outcomes reported represent measures of change in a patient’s status; however, these measures do not show the absolute status of a patient. Ankylosing Spondylitis Disease Activity Score (ASDAS) inactive disease, defined as ASDAS < 1.3, was investigated as a measure of the absolute status of a patient in a limited number of studies. Unfortunately, limited reporting on ASDAS inactive disease among the identified studies prevented assessment of this outcome. Similarly, most studies did not report change in active inflammatory lesions (e.g., short-tau inversion recovery, gadolinium-enhanced T1-weighed imaging), though CRP was reported broadly and was investigated as a marker for change in active inflammation. Another limitation of this analysis was that data collected between weeks 12 to 16 were pooled because of the lack of consistent reporting across studies. Given that ankylosing spondylitis is a chronic disease, it was expected that results at these timepoints would be comparable. Furthermore, safety outcomes such as adverse events, infection, and toxicity were not examined in this study. Since safety events are typically uncommon, RCTs often lack sufficient follow-up and sample size to detect differences in such outcomes. Other NMAs examining adverse events have pooled indications to overcome this limitation [27]. Finally, almost all indications of superior efficacy of GOL IV, IFX, and TOF were in comparison to failed investigational therapies (e.g., UST, APR, RIS) and PBO.

## Conclusions

The SLR and NMA presented in this study are the most up-to-date assessment of the comparative efficacy of current and investigational biologic and OSM therapies for ankylosing spondylitis. Two approved interventions ranked highest among all treatments for efficacy outcomes. Tofacitinib 5 mg was ranked the highest for ASAS20 (though data was derived from a phase 2 study), golimumab IV for change from baseline in BASFI, and infliximab for change from baseline in CRP.

## Electronic supplementary material

ESM 1 (DOCX 832 kb)
