# Supplementary material for: A systematic review and network meta-analysis of current and investigational treatments for active ankylosing spondylitis
Source: Clin Rheumatol. 2020 Feb 27;39(8):2307–15. doi: 10.1007/s10067-020-04970-3 (PMC7338808; doi:10.1007/s10067-020-04970-3)
Supplement: Supplementary file 1 — (DOCX 832 kb) [file 10067_2020_4970_MOESM1_ESM.docx]

**SUPPLEMENTAL MATERIAL: A systematic review and network meta-analysis of current**

**and investigational treatments for active ankylosing spondylitis**

APPENDIX S1. STUDY AND PATIENT CHARACTERISTICS OF THE INCLUDED STUDIES.

APPENDIX S2. PRISMA FLOW DIAGRAM.

APPENDIX S3. DETAILED SEARCH STRATEGY

APPENDIX S4. RISK OF BIAS ASSESSMENT

APPENDIX S5. EVIDENCE NETWORKS

APPENDIX S6. MODEL FIT STATISTICS

APPENDIX S7. RESULTS FROM THE UNADJUSTED MODEL, BASELINE RISKADJUSTED MODEL, AND SENSITIVITY ANALYSIS FOR EACH OUTCOME

APPENDIX S8. INCONSISTENCY ANALYSES

APPENDIX S9: PRISMA-NMA CHECKLIST

APPENDIX S10: HETEROGENEITY ANALYSIS OF PAIRWISE COMPARISONS WITH PLACEBO

**APPENDIX S1. STUDY AND PATIENT CHARACTERISTICS OF THE INCLUDED STUDIES**

| **Study** | **Duration used in the NMA (weeks)** | **Phase** | **Treatment** | **n** | **Age (years)** | **Male (%)** | **RaceWhite**  **(%)** | **HLA-B27 (%)** | **Disease duration (years)** | **Baseline BASDAI (cm)** | **Baseline BASFI (cm)** | **Baseline CRP (mg/l)** | **MTX use (%)** |
| --- | --- | --- | --- | --- | --- | --- | --- | --- | --- | --- | --- | --- | --- |
| **TNF Inhibitors** |  |  |  |  |  |  |  |  |  |  |  |  |  |
| Deodhar 2018 (GO-ALIVE)([1](#_ENREF_1)) | 16 | III | GOL IV 2 mg/kg | 105 | 38.4 (10.1) | 81.9 | NR | 89.9 | 5.6 (6.6)^c^ | 7.0 (1.2) | 6.3 (1.9) | 20.0 (18.2) | 14.3 |
|  |  |  | PBO | 103 | 39.2 (10.8) | 74.8 | NR |  | 5.5 (5.9)^c^ | 7.1 (1.2) | 6.1 (2.0) | 19.3 (16.7) | 20.4 |
| Inman 2008([2](#_ENREF_2)) | 14 | III | GOL SC 50 mg Q4W | 138 | 38.0 (12.6) | 73.9 | 74.6 | 81.8 | 11.0 (9.6) | 6.6 (1.5) | 5.0 (2.6) | 11 (15) | 21 |
|  |  |  | GOL SC 100 mg Q4W | 140 | 38.0 (12.6) | 70.0 | 72.9 | 84.3 | 11.0 (10.0) | 7.0 (1.4) | 5.4 (2.9) | 9 (15.6) | 20 |
|  |  |  | PBO | 78 | 41.0 (14.1) | 70.5 | 73.1 | 84.6 | 16.0 (13.3) | 6.6 (1.5) | 4.9 (2.4) | 11.5 (15.6) | 19.2 |
| Bao 2014([3](#_ENREF_3)) | 16 | III | GOL SC 50 mg Q4W | 108 | 30.5 (10.3) | 83.3 | NR | NR | 4.2 (5.22)^c^ | 6.6 (1.3) | 5.0 (2.4) | 21 (21) | 19.4 |
|  |  |  | PBO | 105 | 30.6 (8.6) | 82.9 | NR | NR | 3.7 (3.9)^c^ | 6.5 (1.5) | 5.0 (2.4) | 19 (20) | 21.9 |
| Braun 2002([4](#_ENREF_4)) | 12 | NR | IFX 5 mg/kg | 34 | 40.6 (8.0) | 68.0 | NR | 91.0 | 16.4 (8.3) | 6.5 (1.2) | 5.4 (1.8) | 24 (21) | Not allowed |
|  |  |  | PBO | 35 | 39.0 (9.1) | 63.0 | NR | 88.0 | 14.9 (9.3) | 6.3 (1.4) | 5.1 (2.2) | 18 (12) | Not allowed |
| van der Heijde 2005 (ASSERT)([5](#_ENREF_5)) | 12 | III | IFX 5 mg/kg | 201 | 40.0 (11.1) | 78.1 | 98 | 86.5 | 7.7 (8.6) | 6.6 (1.7) | 5.7 (1.9) | 15 (19) | Not allowed |
|  |  |  | PBO | 78 | 41.0 (9.6) | 87.2 | 97.4 | 88.5 | 13.2 (10.5) | 6.5 (1.4) | 6.0 (2.3) | 17 (19) | Not allowed |
| van der Heijde 2006 (A)([6](#_ENREF_6)) | 12 | III | ADA 40 mg QOW | 208 | 41.7 (11.7) | 75.5 | NR | 78.4 | 11.3 (10.0) | 6.3 (1.7) | 5.2 (2.2) | 18 (22) | 9.6 |
|  |  |  | PBO | 107 | 43.4 (11.3) | 73.8 | NR | 79.4 | 10.0 (8.3) | 6.3 (1.7) | 5.6 (2.2) | 22 (29) | 7.5 |
| Maksymowych 2008([7](#_ENREF_7)) | 12 | III | ADA 40 mg QOW | 38 | 41.9 (11.1) | 76.3 | NR | 86.8 | 14.5 (9.0)^c^ | 6.2 (1.7) | 5.3 (2.0) | 18 (17) | 10.5 |
|  |  |  | PBO | 44 | 40.0 (10.9) | 81.8 | NR | 81.8 | 12.1 (8.7)^c^ | 6.5 (1.6) | 5.6 (2.2) | 23 (26) | 9.1 |
| Hu 2012([8](#_ENREF_8)) | 12 | NR | ADA 40 mg QOW | 26 | 28.2 (6.9) | 92.3 | NR | 96.2 | 7.4 (5.7) | 5.9 (1.4) | 3.7 (2.1) | 25 (23) | MTX |
|  |  |  | PBO | 20 | 27.4 (7.2) | 100.0 | NR | 95.0 | 7.6 (4.6) | 6.2 (1.1) | 3.9 (2.0) | 32 (29) | MTX |
| Huang 2014([9](#_ENREF_9)) | 12 | III | ADA 40 mg QOW | 229 | 30.1 (8.7) | 80.8 | NR | 95.6 | 3.0 (3.8)^c^ | 6.0 (1.4) | 4.3 (2.3) | 22 (24) | 22.7 |
|  |  |  | PBO | 115 | 29.6 (7.5) | 82.6 | NR | 94.8 | 3.0 (3.2)^c^ | 6.2 (1.4) | 4.4 (2.3) | 23 (30) | 21.7 |
| Landewe 2014([10](#_ENREF_10)) | 12 | III | CZP 200 mg Q2W | 65 | 41.0 (10.8) | 72.3 | NR | 81.5 | 8.8 (5.4)^d^ | 6.5 (1.7) | 5.6 (2.3) | 23 (30) | NR |
|  |  |  | CZP 400 mg Q4W | 56 | 41.9 (11.5) | 73.2 | NR | 78.6 | 8.8 (7.4)^d^ | 6.2 (1.3) | 5.7 (2.3) | 21 (22) | NR |
|  |  |  | PBO | 57 | 41.6 (12.8) | 71.9 | NR | 84.2 | 10.2 (8.4)^d^ | 6.4 (1.9) | 6.0 (2.0) | 22 (17) | NR |
| Gorman 2002([11](#_ENREF_11)) | 16 | NR | ETN 25 mg BIW | 20 | 38.0 (10.0) | 65.0 | 75 | 95.0 | 15.0 (10.0) | N/A | 4.5 (2.1) | 20 (18) | 35 (DMARD) |
|  |  |  | PBO | 20 | 39.0 (10.0) | 90.0 | 70 | 90.0 | 12.0 (9.0) | N/A | 3.2 (2.5) | 15 (12) | 40 (DMARD) |
| Davis 2003([12](#_ENREF_12)) | 12 | II | ETN 25 mg BIW | 138 | 42.1 (11.5) | 76.0 | 94 | 84.0 | 10.1 (7.7)^c^ | 5.8 (1.2) | 5.2 (1.6) | 19 (17) | 11 |
|  |  |  | PBO | 139 | 41.9 (11.8) | 76.0 | 91 | 84.0 | 10.5 (8.8)^c^ | 6.0 (1.2) | 5.6 (1.4) | 20 (17) | 12 |
| Calin 2004([13](#_ENREF_13)) | 12 | III | ETN 25 mg BIW | 45 | 45.3 (9.5) | 80.0 | 93 | NR | 15.0 (8.8) | 6.1 (1.6)^a^ | 6.0 (2.1)^a^ | 19 (16)^a^ | 13 |
|  |  |  | PBO | 39 | 40.7 (11.4) | 77.0 | 95 | NR | 9.7 (8.2) | 5.9 (1.3)^a^ | 5.7 (1.6)^a^ | 24 (42)^a^ | 13 |
| van der Heijde 2006 (B)([14](#_ENREF_14)) | 12 | III | ETN 50 mg QW | 155 | 41.5 (11.0) | 69.7 | NR | NR | 9.0 (8.7) | 6.2 (1.7) | 6.1 (2.0) | 22 (25) | 41.9  (DMARD) |
|  |  |  | ETN 25 mg BIW | 150 | 39.8 (10.7) | 76.0 | NR | NR | 10.0 (9.1) | 5.9 (1.7) | 5.8 (2.0) | 20 (21) | 36.7 (DMARD) |
|  |  |  | PBO | 51 | 40.1 (10.9) | 78.4 | NR | NR | 8.5 (6.8) | 6.1 (1.4) | 6.0 (1.9) | 22 (23) | 33.3 (DMARD) |
| Barkham 2010([15](#_ENREF_15)) | 12 | NR | ETN 25 mg BIW | 20 | 40.8 (9.7) | 75.0 | NR | NR | 11.0 (7.2) | 6.0 (1.7) | 5.6 (2.0) | N/A | MTX |
|  |  |  | PBO | 20 | 39.4 (10.1) | 85.0 | NR | NR | 20.0 (4.9) | 5.5 (1.7) | 5.3 (1.8) | N/A | MTX |
| **IL-12/23 Inhibitors** |  |  |  |  |  |  |  |  |  |  |  |  |  |
| Deodhar 2018 - 3001 (Study 1 - naive to anti-TNF)([16](#_ENREF_16)) | 16 | III | UST 45 mg | 116 | 39.2 (10.5) | 80.2 | 72.4 | 95.7 | 6.3 (7.05)^c^ | 7.4 (1.3) | 6.8 (1.9) | 21.0 (22.1) | 25 |
|  |  |  | UST 90 mg | 114 | 39.5 (11.3) | 87.7 | 71.9 | 96.5 | 6.3 (6.45)^c^ | 7.3 (1.3) | 7.0 (1.6) | 25.0 (31.1) | 19 |
|  |  |  | PBO | 116 | 38.3 (11.4) | 87.1 | 74.1 | 97.4 | 6.6 (6.99)^c^ | 7.4 (1.4) | 6.6 (2.2) | 21.0 (20.8) | 24 |
| Deodhar 2018 - 3002 (Study 2 - refractory to anti-TNF)([16](#_ENREF_16)) | 16 | III | UST 45 mg | 106 | 41.4 (11.3) | 83.0 | 79.2 | 89.6 | 9.1 (7.94)^c^ | 7.6 (1.4) | 6.9 (2.0) | 26.0 (27.0) | 28 |
|  |  |  | UST 90 mg | 105 | 41.5 (11.0) | 87.6 | 80 | 93.3 | 9.6 (8.09)^c^ | 7.5 (1.3) | 6.9 (1.7) | 25.0 (25.0) | 36 |
|  |  |  | PBO | 104 | 40.8 (11.7) | 76.9 | 78.8 | 92.3 | 7.8 (6.73)^c^ | 7.5 (1.3) | 7.2 (1.6) | 28.0 (31.8) | 25 |
| **IL-23 Inhibitors** |  |  |  |  |  |  |  |  |  |  |  |  |  |
| Baeten 2018([17](#_ENREF_17)) | 12 | II | RIS 18 mg | 40 | 38.0 (11.1) | 70.0 | NR | 75.0 | 7.4 (8.2) | 6.4 (1.4) | NR | NR | 20 (DMARD) |
|  |  |  | RIS 90 mg | 39 | 39.5 (10.8) | 77.0 | NR | 77.0 | 6.6 (8.8) | 5.8 (1.6) | NR | NR | 20.5 (DMARD) |
|  |  |  | RIS 180 mg | 40 | 40.6 (11.9) | 75.0 | NR | 85.0 | 10.2 (9.5) | 6.1 (2.2) | NR | NR | 50 (DMARD) |
|  |  |  | PBO | 40 | 37.6 (11.0) | 63.0 | NR | 65.0 | 8.1 (8.2) | 6.3 (1.5) | NR | NR | 50 (DMARD) |
| **IL-17A Inhibitors** |  |  |  |  |  |  |  |  |  |  |  |  |  |
| van der Heijde 2018 (COAST-V)([18](#_ENREF_18)) | 16 | III | ADA 40 mg Q2W | 90 | 41.8 (11.4) | 81.0 | 63 | 91.0 | 7.5 (7.5) | 6.7 (1.5) | 6.1 (2.1) | 12.5 (17.6) | 9 |
|  |  |  | IXE Q2W | 83 | 41.3 (11.2) | 77.0 | 62.6 | 90.0 | 8.2 (9.0) | 6.7 (1.6) | 6.3 (2.1) | 13.4 (15.3) | 5 |
|  |  |  | IXE Q4W | 81 | 41.0 (12.1) | 84.0 | 64.2 | 93.0 | 8.3 (9.6) | 6.8 (1.3) | 6.1 (1.8) | 12.2 (13.3) | 11 |
|  |  |  | PBO | 87 | 42.7 (12.0) | 83.0 | 68.7 | 89.0 | 6.8 (7.6) | 6.8 (1.2) | 6.4 (1.9) | 16.0 (21.0) | 9 |
| Deodhar 2018([19](#_ENREF_19)) | 16 | III | IXE Q2W | 98 | 44.2 (10.8) | 76.5 | 79.6 | NR | 11.7 (8.8) | 7.5 (1.3) | 7.4 (1.4) | 16.9 (19.8) | 9.2 |
|  |  |  | IXE Q4W | 114 | 47.4 (13.4) | 79.8 | 80.5 | NR | 10.1 (7.8) | 7.5 (1.3) | 7.4 (1.8) | 20.2 (34.3) | 10.5 |
|  |  |  | PBO | 104 | 46.6 (12.7) | 83.7 | 81.7 | NR | 13.0 (10.5) | 7.3 (1.3) | 7.0 (1.7) | 16.0 (22.3) | 19.2 |
| Deodhar 2016 (MEASURE 1)([20](#_ENREF_20)) | 16 | III | SEC IV 75 mg | 124 | 42.3 (13.2) | 71.0 | 61.3 | 79.8 | 7.9 (9.7)^c^ | 6.0 (1.4) | 5.4 (2.2) | NR | NR |
|  |  |  | SEC IV 150 mg | 125 | 40.1 (11.6) | 67.2 | 55.2 | 68.8 | 6.5 (6.9)^c^ | 6.3 (1.6) | 5.6 (2.2) | NR | NR |
|  |  |  | PBO | 122 | 43.1 (12.4) | 69.7 | 66.4 | 73.8 | 8.3 (8.9)^c^ | 6.5 (1.5) | 5.8 (2.0) | NR | NR |
| Marzo-Ortega 2017 (MEASURE 2)([21](#_ENREF_21)) | 16 | III | SEC SC 75 mg | 73 | 44.4 (13.1) | 69.9 | NR | 72.6 | 5.3 (7.4)^c^ | 6.6 (1.3) | NR | 5.7 (0.5-86.2)^b^ | MTX |
|  |  |  | SEC SC 150 mg | 72 | 41.9 (12.5) | 63.9 | NR | 79.2 | 7.0 (8.2)^c^ | 6.6 (1.5) | NR | 7.5 (0.4-237.0)^b^ | MTX |
|  |  |  | PBO | 74 | 43.6 (13.2) | 75.7 | NR | 78.4 | 6.4 (8.9)^c^ | 6.8 (1.3) | NR | 8.3 (0.5-84.6)^b^ | MTX |
| Pavelka 2017 (MEASURE 3)([22](#_ENREF_22)) | 16 | III | SEC IV 150 mg | 74 | 42.9 (11.1) | 62.2 | 73 | 70.3 | 6.0 (7.2)^c^ | 7.0 (1.4) | NR | 21.1 (0.4-111.3)^b^ | 13.5 |
|  |  |  | SEC IV 300mg | 76 | 42.1 (11.8) | 65.8 | 68.4 | 73.7 | 5.3 (7.3)^c^ | 7.0 (1.4) | NR | 13.3 (0.2-65.1)^b^ | 17.1 |
|  |  |  | PBO | 76 | 42.7 (11.4) | 52.6 | 76.3 | 69.7 | 5.2 (6.4)^c^ | 6.9 (1.3) | NR | 20.0 (0.2-112.5)^b^ | 7.9 |
| Kivitiz 2018 (MEASURE 4)([23](#_ENREF_23)) | 16 | III | SEC 150 mg | 116 | 44.5 (11.6) | 69.8 | 97.4 | 86.2 | 8.4 (10.84)^c^ | 7.0 (1.2) | NR | 6.25 (0.4-123.0)^b^ | 9.5 |
|  |  |  | SEC 150 mg - no loading dose | 117 | 41.2 (11.1) | 70.9 | 100 | 84.6 | 6.5 (7.55)^c^ | 7.0 (1.3) | NR | 6.20 (0.3-120.9)^b^ | 9.4 |
|  |  |  | PBO | 117 | 43.4 (12.5) | 65.0 | 97.4 | 79.5 | 7.1 (9.23)^c^ | 7.1 (1.3) | NR | 5.40 (0.3-129.3)^b^ | 8.5 |
| **JAK Inhibitors** |  |  |  |  |  |  |  |  |  |  |  |  |  |
| van der Heijde 2018 (TORTUGA)([24](#_ENREF_24)) | 12 | II | FIL 200 mg | 58 | 41.0 (11.6) | 78.0 | NR | 88.0 | 6.0 (5.5) | 6.9 (1.2) | 7.0 (1.5) | 19.6 (13.3) | 16 |
|  |  |  | PBO | 58 | 42.0 (9.0) | 71.0 | NR | 88.0 | 8.0 (7.6) | 7.0 (1.3) | 6.9 (1.6) | 21.2 (23.0) | 7 |
| van der Heijde 2017([25](#_ENREF_25)) | 12 | II | TOF 10 mg BID | 52 | 41.6 (12.2) | 73.1 | NR | 94.2 | NR | 6.9 (1.7) | 5.7 (2.4) | NR | 30.8 (csDMARD) |
|  |  |  | TOF 5 mg BID | 52 | 41.2 (10.3) | 75.0 | NR | 84.6 | NR | 6.5 (1.9) | 5.8 (2.2) | NR | 30.8 (csDMARD) |
|  |  |  | TOF 2 mg BID | 52 | 41.8 (12.3) | 65.4 | NR | 84.6 | NR | 7.0 (1.7) | 5.5 (1.9) | NR | 44.2 (csDMARD) |
|  |  |  | PBO | 51 | 41.9 (12.9) | 62.7 | NR | 86.3 | NR | 6.3 (1.9) | 5.7 (2.3) | NR | 27.5  (csDMARD) |
| **PDE4 Inhibitors** |  |  |  |  |  |  |  |  |  |  |  |  |  |
| Pathan 2012([26](#_ENREF_26)) | 12 | II | APR 30 mg BID | 17 | 44.9 (11.1) | NR | NR | NR | 20.9 (12.3) | 4.8 (2.2) | 4.6 (2.4) | 11.4 (12.1) | Not allowed |
|  |  |  | PBO | 19 | 39.2 (13.3) | NR | NR | NR | 18.4 (10.2) | 4.4 (1.8) | 3.5 (2.2) | 6.2 (2.6) | Not allowed |
| Unpublished (NCT01583374)([27](#_ENREF_27)) | 16 | III | APR 20 mg | 163 | 45.2 (11.9) | 74.2 | 94.5 | NR | 11.1 (11.3)^c^ | NR | NR | NR | NR |
|  |  |  | APR 30 mg | 163 | 44.8 (11.8) | 65.6 | 96.9 | NR | 10.3 (9.9)^c^ | NR | NR | NR | NR |
|  |  |  | PBO | 164 | 44.0 (12.9) | 75.6 | 96.3 | NR | 10.4 (10.4)^c^ | NR | NR | NR | NR |

Note: Values are mean (SD) unless otherwise specified

^a^ Data extracted from Wang et al. ^3^

^b^ Studies reported hsCRP median (min-max)

^c^ Studies labelled disease duration using synonyms such as Time Since Diagnosis of AS, AS Diagnosis, Duration of AS, and Duration of Disease Since Axial Sponyloarthritis diagnosis

^d^ Based on Symptom Duration

Abbreviations: ADA = adalimumab; APR = apremilast; BASDAI = Bath Ankylosing Spondylitis Disease Activity Index; BASFI = Bath Ankylosing Spondylitis Functional Index; BID = twice a day; BIW = biweekly; CRP = C-reactive protein; csDMARD = conventional synthetic disease-modifying antirheumatic drug; CZP = certolizumab pegol; DMARD = disease-modifying antirheumatic drugs; ETN = etanercept; FIL = filgotinib; GOL = golimumab; HLA-B27 = human leukocyte antigen B27; hs-CRP = highly sensitive C-reactive protein; IFX = infliximab; IL = interleukin; IV = intravenous; IXE = ixekizumab; JAK = Janus Kinase; MTX = methotrexate; NMA = network meta-analysis; NR = not reported; PBO = placebo; PDE = phosphodiesterase; QW = once per week; QOW= every other week; Q2W = every 2 weeks; Q4W = every 4 weeks; RIS = risankizumab; SEC = secukinumab; SC = subcutaneous; TNF = tumor necrosis factor; TOF = tofacitinib; UST = ustekinumab.

**APPENDIX S2. PRISMA FLOW DIAGRAM. Abbreviations: MA = meta-analysis; SLR = systematic literature review.**


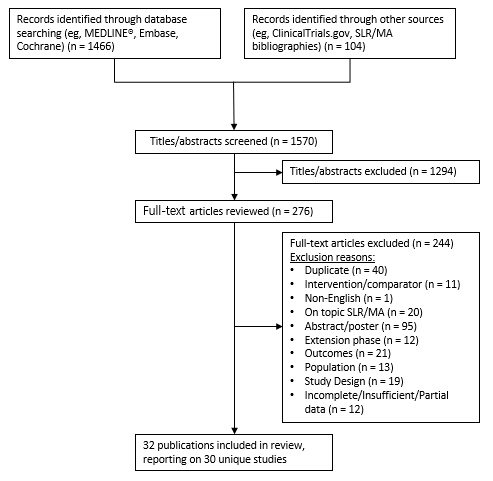


**ADDPENDIX S3. DETAILED SEARCH STRATEGY**

MEDLINE

Database: Ovid MEDLINE(R) Epub Ahead of Print, In-Process & Other Non-Indexed Citations, Ovid MEDLINE(R) Daily and Ovid MEDLINE(R) <1946 to Present>

Search Strategy:

--------------------------------------------------------------------------------

1 Spondylitis, Ankylosing/ (13663)

2 ((ankylosing or ankylosans or ankylos#s or ankylotic or ankylopoietic or ankylopoietica or rheumatoid) adj2 (spondilit#s or spondylarthrit* or spondylarthros#s or spondyloarthrit* or spondylit#s or spinal or spine or vertebral)).tw,kf. (14096)

3 Bechterew*.tw,kf. (569)

4 Marie-Struempell*.tw,kf. (7)

5 or/1-4 [Ankylosing Spondylitis] (17850)

6 (controlled clinical trial or randomized controlled trial).pt. (550187)

7 clinical trials as topic.sh. (183889)

8 Randomized Controlled Trials as Topic/ (116750)

9 (randomi#ed or randomi#ation? or randomly or RCT? or placebo*).tw,kf. (855964)

10 ((singl* or doubl* or trebl* or tripl*) adj (mask* or blind* or dumm*)).tw,kf. (157305)

11 trial.ti. (183350)

12 or/6-11 (1256339)

13 5 and 12 [ANKYLOSING SPONDYLITIS RCTs] (1331)

14 exp Animals/ not (exp Animals/ and Humans/) (4464699)

15 13 not 14 [ANIMAL-ONLY REMOVED] (1328)

16 (2016* or 2017* or 2018*).dt. (2926017)

17 15 and 16 [UPDATE PERIOD] (168)

***************************

Embase

Database: Embase <1988 to 2018 Week 22>

Search Strategy:

--------------------------------------------------------------------------------

1 ankylosing spondylitis/ (20230)

2 ((ankylosing or ankylosans or ankylos#s or ankylotic or ankylopoietic or ankylopoietica or rheumatoid) adj2 (spondilit#s or spondylarthrit* or spondylarthros#s or spondyloarthrit* or spondylit#s or spinal or spine or vertebral)).tw,kw. (17990)

3 Bechterew*.tw,kw. (284)

4 Marie-Struempell*.tw,kw. (0)

5 or/1-4 [Ankylosing Spondylitis] (23096)

6 randomized controlled trial/ (485910)

7 controlled clinical trial/ (460080)

8 exp "clinical trial (topic)"/ (268120)

9 (randomi#ed or randomi#ation? or randomly or RCT? or placebo*).tw,kw. (1132350)

10 ((singl* or doubl* or trebl* or tripl*) adj (mask* or blind* or dumm*)).tw,kw. (188206)

11 trial.ti. (228738)

12 or/6-11 (1613464)

13 5 and 12 [Ankylosing Spondylitis RCTs] (2660)

14 exp animal/ or exp animal experimentation/ or exp animal model/ or exp animal experiment/ or nonhuman/ or exp vertebrate/ (21278367)

15 exp human/ or exp human experimentation/ or exp human experiment/ (16607079)

16 14 not 15 (4671828)

17 13 not 16 [ANIMAL-ONLY REMOVED] (2646)

18 (2016* or 2017* or 2018*).dc. (4005654)

19 17 and 18 (739)

***************************

Cochrane Library

Search Name: Ankylosing Spondylitis - Update

Date Run: 28/05/18 18:16:56.174

Description: Final - 2018 May 28

ID Search Hits

#1 [mh "Spondylitis, Ankylosing"] 548

#2 ((ankylosing or ankylosans or ankylosis or ankyloses or ankylotic or ankylopoietic or ankylopoietica or rheumatoid) near/2 (spondilit* or spondylarthrit* or spondylarthros* or spondyloarthrit* or spondylit* or spinal or spine or vertebral)):ti,ab,kw 1445

#3 Bechterew*:ti,ab,kw 14

#4 (Marie next Struempell*):ti,ab,kw 0

#5 ([28-#4](#_ENREF_28)) Publication Year from 2016 to 2018 387

CENTRAL – 386 [RCTs]

***************************

**APPENDIX S4.** **RISK OF BIAS ASSESSMENT**

| Study | Random sequence generation | Allocation concealment | Blinding of participants and personnel | Blinding of outcomes assessed | Incomplete outcome data | Selective reporting |
| --- | --- | --- | --- | --- | --- | --- |
| Deodhar 2018 (GO-ALIVE)* |  |  |  |  |  |  |
| Inman 2008* |  |  |  |  |  |  |
| Bao 2014* |  |  |  |  |  |  |
| Braun 2002 |  |  |  |  |  |  |
| van der Heijde 2005 (ASSERT) |  |  |  |  |  |  |
| van der Heijde 2006 (A)* |  |  |  |  |  |  |
| Maksymowych 2008* |  |  |  |  |  |  |
| Hu 2012 |  |  |  |  |  |  |
| Huang 2014 |  |  |  |  |  |  |
| Landewe 2014* |  |  |  |  |  |  |
| Gorman 2002 |  |  |  |  |  |  |
| Davis 2003 |  |  |  |  |  |  |
| Calin 2004 |  |  |  |  |  |  |
| van der Heijde 2006 (B) |  |  |  |  |  |  |
| Barkham 2010 |  |  |  |  |  |  |
| Baeten 2018 |  |  |  |  |  |  |
| Deodhar 2018 - 3001 (Study 1 - naive to anti-TNF) |  |  |  |  |  |  |
| Deodhar 2018 - 3002 (Study 2 - refractory to anti-TNF) |  |  |  |  |  |  |
| van der Heijde 2018 (COAST-V) |  |  |  |  |  |  |
| Deodhar 2018 |  |  |  |  |  |  |
| Deodhar 2016 (MEASURE 1)* |  |  |  |  |  |  |
| Marzo-Ortega 2017 (MEASURE 2)* |  |  |  |  |  |  |
| Pavelka 2017 (MEASURE 3)* |  |  |  |  |  |  |
| Kivitiz 2018 (MEASURE 4) |  |  |  |  |  |  |
| van der Heijde 2018 (TORTUGA) |  |  |  |  |  |  |
| van der Heijde 2017 |  |  |  |  |  |  |
| Pathan 2012 |  |  |  |  |  |  |
| Unpublished (NCT01583374)** |  |  |  |  |  |  |

Key: low risk of bias; unclear risk of bias; high risk of bias.

*Crossover was allowed in these studies. However, the results used in this analysis were obtained before crossover and thus were not biased by the study design.

**There was no publication for this study. Data was collected from clinicaltrials.gov which did not report full risk of bias information.

**APPENDIX S5. EVIDENCE NETWORKS**

Each intervention is represented by a node and randomized comparisons are shown as links between the nodes. The size of each node represents the number of patients randomized to each treatment.

1. **Change in BASFI NMA**


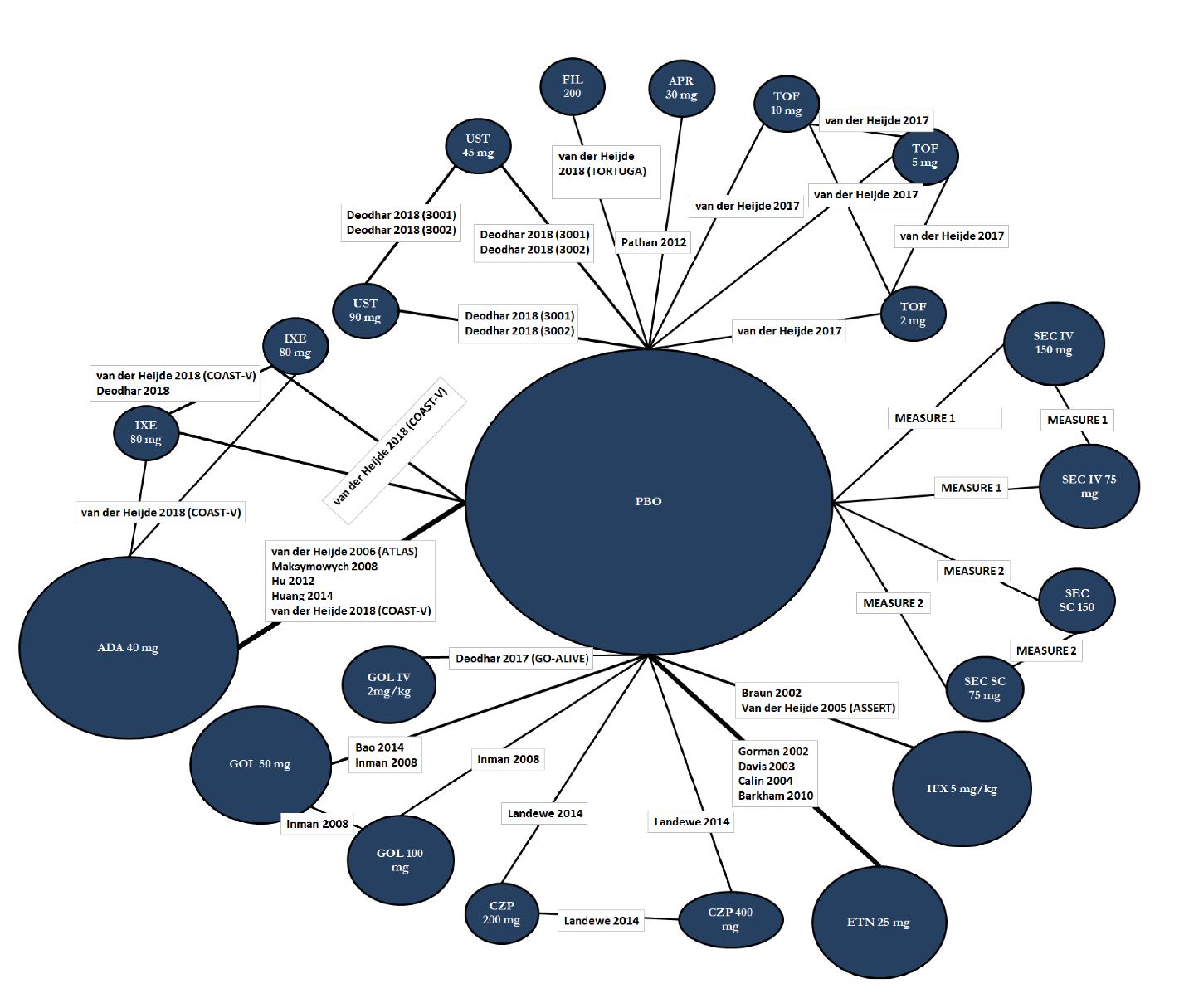


1. **Change in CRP NMA**


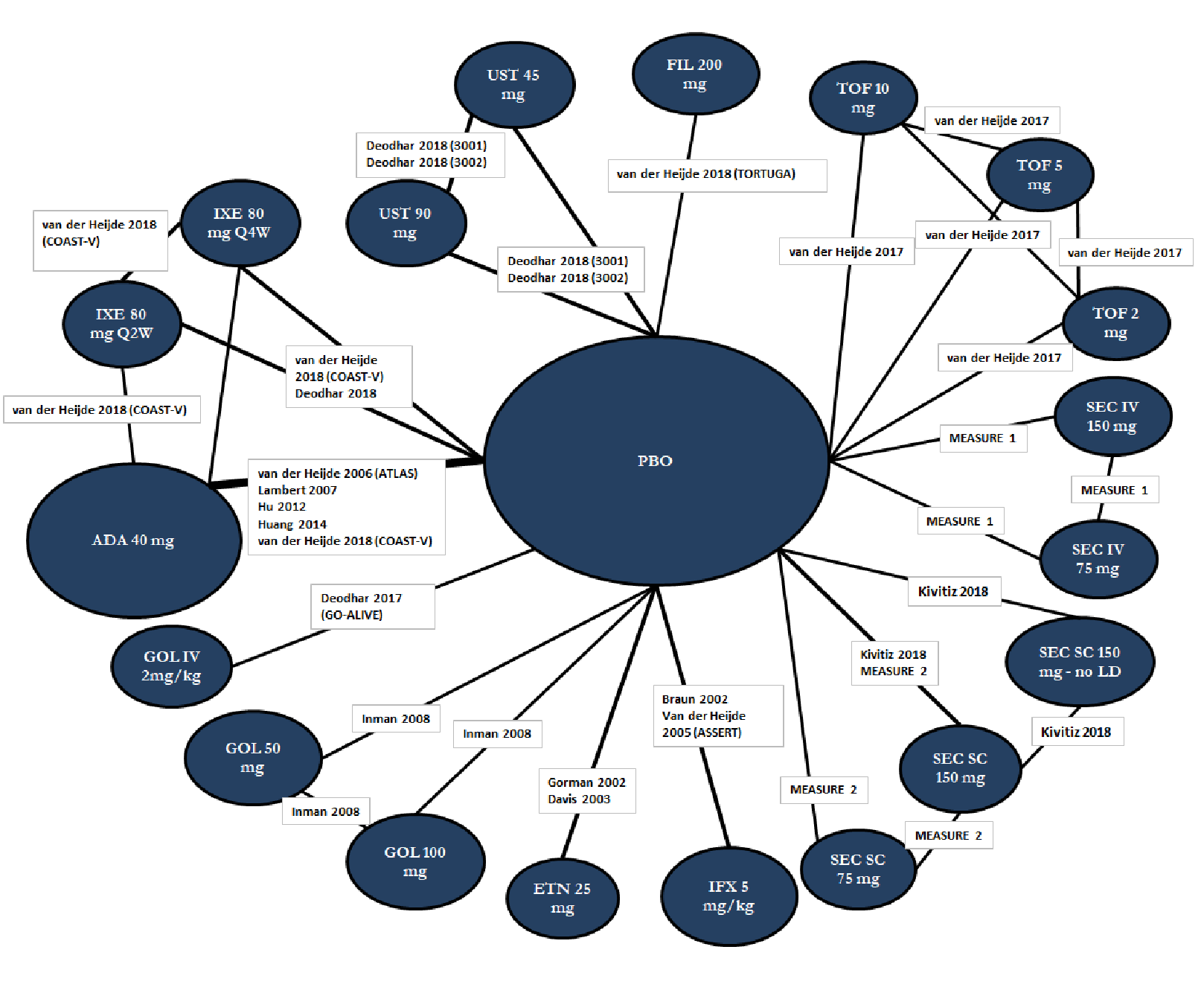


Abbreviations: ADA = adalimumab; ARP = apremilast; CTZ = certolizumab; ETN = etanercept; FIL = filgotinib; GOL = golimumab; IFX = infliximab; IV = intravenous; IXE = ixekizumab; kg = kilogram; LD = loading dose; mg = milligram; PBO = placebo; RIS = risankizumab; TOF = tofacitinib; SC = subcutaneous; SEC = secukinumab; UST = ustekinumab.

**APPENDIX S6. MODEL FIT STATISTICS**

The table below summarizes the model fit statistics for the unadjusted and baseline risk-adjusted NMAs. Baseline risk adjustments accounted for differences in the placebo response across trials. The model with the best fit for each outcome is shaded in grey.

|  | **ASAS20** | **Change in BASFI** | **Change in CRP** |
| --- | --- | --- | --- |
| Unadjusted NMA | Beta: NA  0.29 (0.06 to 0.6)  Res dev: 68.47 vs. 68  DIC: 444.85 | Beta: NA  SD: 0.57 (0.13 to 1.14)  Res dev: 36.82 vs. 34  DIC: 62.4 | Beta: NA  SD: 0.42 (0.14 to 0.9)  Res dev: 28.82 vs. 30  DIC: 45.5 |
| Baseline risk-adjusted NMA | Beta: -0.6 (-1.07 to -0.11)  SD: 0.25 (0.04 to 0.51)  Res dev: 69.3 vs. 68  DIC: 446.9 | Beta: 0.12 (-0.39 to 0.64)  SD: 0.62 (0.16 to 1.25)  Res dev: 36.2 vs. 34  DIC: 62.42 | Beta: -0.01 (-0.56 to 0.66)  SD: 0.43 (0.11 to 0.95)  Res dev: 30.12 vs. 31  DIC: 48.0 |

Abbreviations: ASAS20 = improvement of ≥20% in the Assessment of Spondyloarthritis International Society Criteria; BASFI = Bath Ankylosing Spondylitis Functional Index; CRP = C-reactive protein; DIC = deviance information criterion; NA = not applicable; NMA = network meta-analysis; res dev = residual deviation; SD = standard deviation.

**APPENDIX S7. RESULTS FROM THE UNADJUSTED MODEL, BASELINE RISK-ADJUSTED MODEL, AND SENSITIVITY ANALYSIS FOR EACH OUTCOME**

The tables below provide results from the unadjusted model, baseline risk-adjusted model, and sensitivity analysis that included 10-week studies for the ASAS20, change in BASFI, and change in CRP NMAs. Results show pairwise comparisons for GOL IV 2 mg/kg vs. comparators for each analysis.

**Results from the ASAS20 NMAs**

| **Golimumab IV 2 mg/kg vs. Comparator** | **Unadjusted**  **ASAS20**  **OR (95% CrIs)** | **Adjusted**  **ASAS20**  **OR (95% CrIs)** | **Sensitivity Analysis ASAS20**  **OR (95% CrIs)** |
| --- | --- | --- | --- |
| Placebo | **7.9 (3.21 to 19.87)** | **3.55 (1.32 to 9.33)** | **3.64 (1.38 to 9.49)** |
| Adalimumab 40 mg | 2.26 (0.83 to 6.33) | 2.06 (0.91 to 4.84) | 2.03 (0.95 to 4.71) |
| Apremilast 30 mg | **7.75 (2.37 to 23.04)** | **6.34 (2.37 to 16.36)** | **6.44 (2.5 to 16.54)** |
| Certolizumab 200 mg | 3.44 (0.92 to 13.65) | 2.55 (0.84 to 7.94) | 2.62 (0.87 to 7.76) |
| Certolizumab 400 mg | 2.5 (0.66 to 9.74) | 1.88 (0.58 to 5.82) | 1.85 (0.62 to 5.88) |
| Etanercept 25 mg | 1.74 (0.6 to 4.78) | 1.56 (0.65 to 3.63) | 1.56 (0.69 to 3.63) |
| Etanercept 50 mg | 1.54 (0.45 to 5.27) | 1.26 (0.45 to 3.44) | 1.4 (0.57 to 3.53) |
| Filgotinib 200 mg | 1.63 (0.4 to 6.44) | 1.12 (0.35 to 3.56) | 1.13 (0.35 to 3.51) |
| Golimumab SC 100 mg | 1.74 (0.5 to 5.84) | 1.89 (0.67 to 5.07) | 1.82 (0.69 to 4.99) |
| Golimumab SC 50 mg | 1.99 (0.65 to 6.16) | 2.23 (0.89 to 5.52) | 2.17 (0.91 to 5.3) |
| Infliximab 5 mg/kg | 1.19 (0.37 to 3.84) | 1.41 (0.53 to 3.57) | 1.53 (0.64 to 3.74) |
| Ixekizumab 80 mg Q2W | 2.55 (0.85 to 7.77) | 2.29 (0.9 to 5.72) | 2.28 (0.93 to 5.46) |
| Ixekizumab 80 mg Q4W | 2.74 (0.93 to 8.1) | 2.47 (0.97 to 5.95) | 2.41 (0.98 to 5.85) |
| Risankizumab 18 mg single dose | 2.38 (0.5 to 10.32) | 2.93 (0.84 to 9.68) | 2.85 (0.91 to 9.24) |
| Risankizumab 180 mg | **4.59 (1.02 to 20.66)** | **5.73 (1.6 to 19.18)** | **5.61 (1.73 to 20.3)** |
| Risankizumab 90 mg | 3.93 (0.85 to 17.74) | **4.85 (1.45 to 15.63)** | **4.78 (1.29 to 16.0)** |
| IV Secukinumab 150 mg | 2.49 (0.83 to 7.42) | 2.12 (0.85 to 5.48) | 2.33 (0.99 to 5.55) |
| IV Secukinumab 300 mg | 2.63 (0.74 to 9.04) | 2.14 (0.72 to 6.16) | 2.18 (0.78 to 6.17) |
| Secukinumab IV 75 mg | 2.35 (0.69 to 8.19) | 2.06 (0.76 to 5.74) | 2.12 (0.83 to 5.74) |
| Secukinumab SC 150 mg - no loading dose | **3.7 (1.1 to 12.35)** | 2.38 (0.79 to 6.72) | 2.39 (0.85 to 6.57) |
| Secukinumab SC 150 mg | **3.32 (1.1 to 9.81)** | 2.35 (0.88 to 6.05) | 1.83 (0.66 to 5.33) |
| Secukinumab SC 75 mg | **5.96 (1.68 to 20.35)** | **4.63 (1.59 to 13.74)** | **4.15 (1.51 to 12.25)** |
| Tofacitinib 10 mg | **4.37 (1.13 to 17.74)** | 2.93 (0.89 to 9.63) | 2.92 (0.93 to 9.16) |
| Tofacitinib 2 mg | **5.07 (1.32 to 20.74)** | **3.4 (1.03 to 10.74)** | **3.35 (1.1 to 11.06)** |
| Tofacitinib 5 mg | 1.28 (0.31 to 5.6) | 0.84 (0.24 to 2.9) | 0.84 (0.25 to 2.89) |
| Ustekinumab 45 mg | **7.75 (2.62 to 23.68)** | **6.19 (2.49 to 15.9)** | **6.03 (2.58 to 15.41)** |
| Ustekinumab 90 mg | **6.18 (2.04 to 18.59)** | **4.97 (1.98 to 12.75)** | **4.93 (2 to 12.11)** |

Note: Bolded values represent comparisons for which GOL IV 2 mg/kg was superior to the comparator.

Abbreviations: ASAS20 = improvement of ≥20% in the Assessment of Spondyloarthritis International Society Criteria; Crls = credible intervals; IV = intravenous; kg = kilogram; mg = milligram; OR = odds ratio; SC = subcutaneous.

**Results from the change in BASFI NMAs**

| **Golimumab IV 2 mg/kg vs. Comparator** | **Unadjusted**  **Change in BASFI**  **MD (95% CrIs)** | **Adjusted**  **Change in BASFI**  **MD (95% CrIs)** | **Sensitivity Analysis Change in BASFI**  **MD (95% CrIs)** |
| --- | --- | --- | --- |
| Placebo | **-1.9 (-3.34 to -0.49)** | **-1.98 (-3.59 to -0.46)** | **-1.9 (-3.22 to -0.6)** |
| Adalimumab 40 mg | -0.74 (-2.35 to 0.82) | -0.84 (-2.65 to 0.85) | -0.73 (-2.22 to 0.69) |
| Apremilast 30 mg | -0.43 (-2.67 to 1.77) | -0.68 (-3.4 to 1.9) | -0.41 (-2.5 to 1.63) |
| Certolizumab 200 mg | -0.92 (-2.93 to 1.16) | -0.96 (-3.18 to 1.23) | -0.9 (-2.82 to 0.97) |
| Certolizumab 400 mg | -0.8 (-2.81 to 1.27) | -0.84 (-3.1 to 1.38) | -0.79 (-2.68 to 1.12) |
| Etanercept 25 mg | -1.27 (-2.82 to 0.51) | -1.34 (-3.12 to 0.56) | -1.3 (-2.76 to 0.38) |
| Filgotinib 200 mg | -0.68 (-2.77 to 1.38) | -0.6 (-2.85 to 1.67) | -0.67 (-2.59 to 1.2) |
| Golimumab SC 100 mg | -0.35 (-2.37 to 1.59) | -0.51 (-2.79 to 1.68) | -0.34 (-2.15 to 1.46) |
| Golimumab SC 50 mg | -0.44 (-2.18 to 1.3) | -0.58 (-2.63 to 1.33) | -0.42 (-2.03 to 1.15) |
| Infliximab 5 mg/kg | -0.1 (-1.88 to 1.73) | -0.17 (-2.09 to 1.8) | -0.08 (-1.73 to 1.54) |
| Ixekizumab 80 mg every 4 weeks | -0.7 (-2.39 to 1.01) | -0.62 (-2.53 to 1.35) | -0.72 (-2.28 to 0.87) |
| Ixekizumab 80 mg every other week | -0.59 (-2.31 to 1.17) | -0.57 (-2.44 to 1.33) | -0.59 (-2.16 to 1.03) |
| IV Secukinumab 150 mg | -0.48 (-2.53 to 1.56) | -0.58 (-2.77 to 1.63) | -0.49 (-2.37 to 1.36) |
| IV Secukinumab 75 mg | -0.59 (-2.58 to 1.44) | -0.69 (-2.86 to 1.51) | -0.61 (-2.43 to 1.2) |
| Secukinumab SC 150 mg | -0.4 (-2.44 to 1.6) | -0.46 (-2.61 to 1.71) | -0.34 (-2.14 to 1.5) |
| Secukinumab SC 75 mg | -0.89 (-2.94 to 1.1) | -0.98 (-3.19 to 1.18) | -0.8 (-2.41 to 0.91) |
| Tofacitinib 10 mg | -1.12 (-3.19 to 0.97) | -1.17 (-3.38 to 1.06) | -1.13 (-3.07 to 0.87) |
| Tofacitinib 2 mg | -1.38 (-3.5 to 0.69) | -1.46 (-3.69 to 0.78) | -1.37 (-3.36 to 0.54) |
| Tofacitinib 5 mg | -0.88 (-2.99 to 1.2) | -0.95 (-3.28 to 1.23) | -0.87 (-2.86 to 1.03) |
| Ustekinumab 45 mg | **-1.75 (-3.5 to -0.03)** | -1.7 (-3.59 to 0.26) | **-1.77 (-3.4 to -0.16)** |
| Ustekinumab 90 mg | -1.59 (-3.35 to 0.12) | -1.47 (-3.49 to 0.61) | -1.62 (-3.24 to 0.04) |

Note: Bolded values represent comparisons for which GOL IV 2 mg/kg was superior to the comparator.

Abbreviations: BASFI = Bath Ankylosing Spondylitis Functional Index; Crls = credible intervals; IV = intravenous; kg = kilogram; MD = mean difference; mg = milligram; SC = subcutaneous.

**Results from the change in CRP NMAs**

| **Golimumab IV 2 mg/kg vs. Comparator** | **Unadjusted**  **Change in CRP**  **MD (95% CrIs)** | **Adjusted**  **Change in CRP**  **MD (95% CrIs)** | **Sensitivity Analysis Change in CRP**  **MD (95% CrIs)** |
| --- | --- | --- | --- |
| Placebo | **-1.5 (-2.55 to -0.41)** | **-1.5 (-2.59 to -0.35)** | **-1.5 (-2.53 to -0.45)** |
| Adalimumab 40 mg | -0.3 (-1.49 to 0.93) | -0.3 (-1.58 to 0.97) | -0.3 (-1.47 to 0.88) |
| Etanercept 25 mg | -0.32 (-1.69 to 1.17) | -0.33 (-1.7 to 1.19) | -0.33 (-1.65 to 1.12) |
| Filgotinib 200 mg | -0.63 (-2.21 to 0.93) | -0.63 (-2.27 to 0.99) | -0.63 (-2.16 to 0.9) |
| Golimumab SC 100 mg | -0.79 (-2.25 to 0.72) | -0.78 (-2.37 to 0.76) | -0.8 (-2.28 to 0.68) |
| Golimumab SC 50 mg | -0.74 (-2.21 to 0.8) | -0.74 (-2.31 to 0.83) | -0.75 (-2.2 to 0.73) |
| Infliximab 5 mg/kg | 0.31 (-1.19 to 1.89) | 0.05 (-1.3 to 1.52) | 0.06 (-1.22 to 1.4) |
| Ixekizumab 80 mg Q2W | -0.25 (-1.58 to 1.14) | -0.21 (-1.6 to 1.15) | -0.25 (-1.55 to 1.09) |
| Ixekizumab 80 mg Q4W | -0.2 (-1.49 to 1.16) | -0.17 (-1.63 to 1.22) | -0.19 (-1.45 to 1.14) |
| IV Secukinumab 150 mg | -1.05 (-2.98 to 0.87) | -1.08 (-3.02 to 0.89) | -1.07 (-3.01 to 0.88) |
| IV Secukinumab 75 mg | -1.04 (-2.95 to 0.9) | -1.05 (-2.94 to 0.94) | -0.98 (-2.88 to 0.85) |
| Secukinumab SC 150 mg | -1.03 (-2.42 to 0.41) | -1.08 (-2.66 to 0.69) | -1.02 (-2.43 to 0.36) |
| Secukinumab SC 150 mg - no loading dose | -0.77 (-2.25 to 0.73) | -0.87 (-2.74 to 1.38) | -0.77 (-2.22 to 0.67) |
| Secukinumab SC 75 mg | -1.14 (-2.83 to 0.6) | -1.2 (-2.96 to 0.62) | -1.14 (-2.85 to 0.57) |
| Tofacitinib 10 mg | -0.8 (-2.37 to 0.83) | -0.8 (-2.4 to 0.86) | -0.79 (-2.3 to 0.71) |
| Tofacitinib 2 mg | -1.01 (-2.56 to 0.59) | -1 (-2.6 to 0.59) | -1 (-2.51 to 0.54) |
| Tofacitinib 5 mg | -0.9 (-2.47 to 0.66) | -0.9 (-2.5 to 0.74) | -0.9 (-2.4 to 0.62) |
| Ustekinumab 45 mg | -1.29 (-2.64 to 0.11) | -1.3 (-2.72 to 0.17) | -1.29 (-2.62 to 0.04) |
| Ustekinumab 90 mg | -1.07 (-2.47 to 0.29) | -1.09 (-2.57 to 0.33) | -1.08 (-2.47 to 0.21) |

Note: Bolded values represent comparisons for which GOL IV 2 mg/kg was superior to the comparator.

Abbreviations: Crls = credible intervals; CRP = C-reactive protein; IV = intravenous; kg = kilogram; MD = mean difference; mg = milligram; SC = subcutaneous.

**APPENDIX S8. INCONSISTENCY ANALYSIS**

Comparison of posterior deviance and model fit statistics in the Consistency and Inconsistency NMAs.

1. **ASAS20: Adjusted NMA**

**
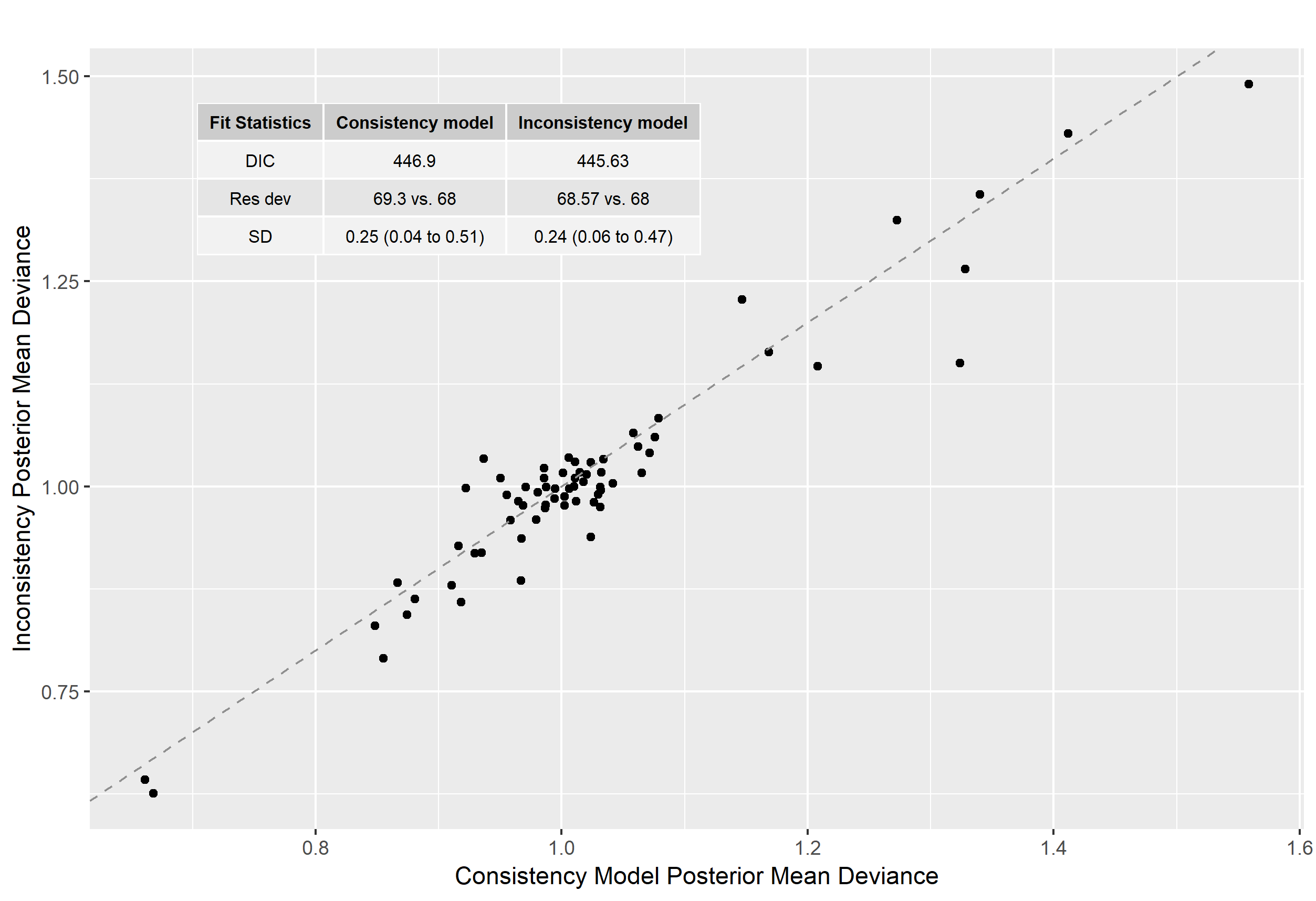
**

Abbreviations: ASAS20 = improvement of ≥20% in the Assessment of Spondyloarthritis International Society Criteria; DIC = deviance information criterion; NMA = network meta-analysis; res dev = residual deviation; SD = standard deviation.

1. **BASFI: Unadjusted NMA**


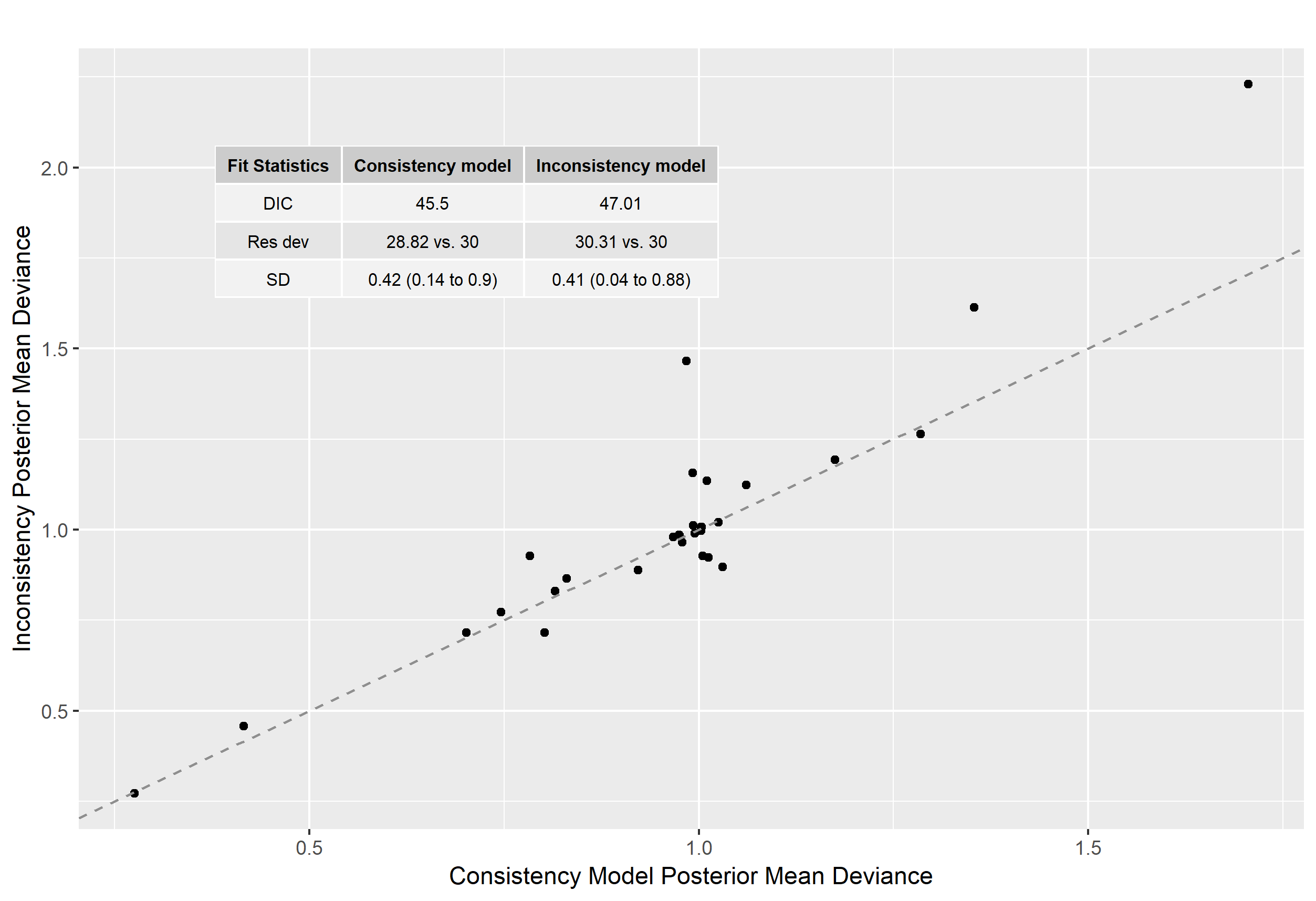


Abbreviations: BASFI = Bath Ankylosing Spondylitis Functional Index; DIC = deviance information criterion; NMA = network meta-analysis; res dev = residual deviation; SD = standard deviation.

1. **CRP: Unadjusted NMA**

**
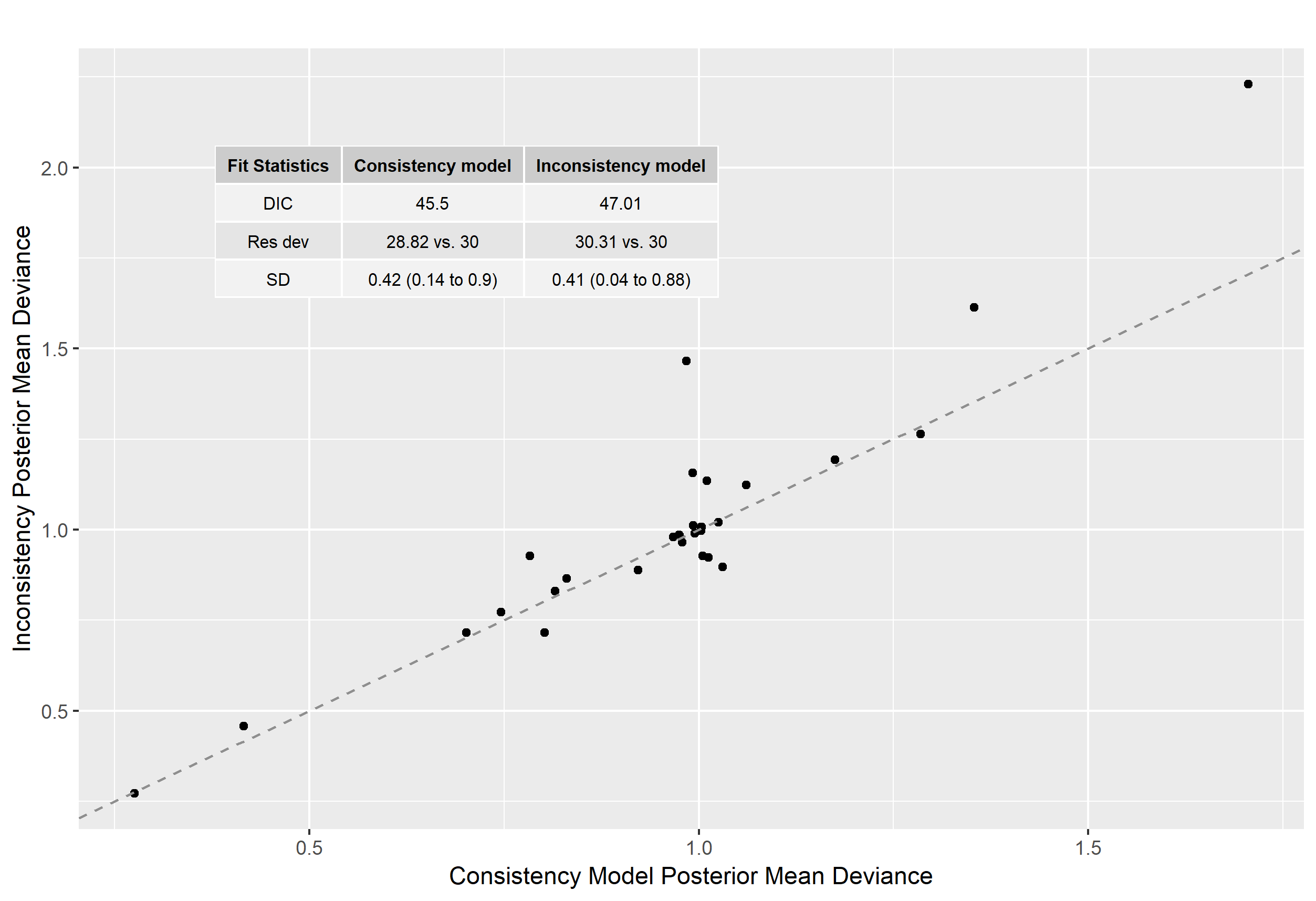
**

Abbreviations: CRP = C-reactive protein; DIC = deviance information criterion; NMA = network meta-analysis; res dev = residual deviation; SD = standard deviation.

**APPENDIX S9. PRISMA-NMA CHECKLIST**

| Section/Topic | Item # | Checklist Item | Reported on Page # |
| --- | --- | --- | --- |
| **TITLE** |  |  |  |
| Title | 1 | Identify the report as a systematic review incorporating a network meta-analysis (or related form of meta-analysis). | 1 |
|  |  |  |  |
| **ABSTRACT** |  |  |  |
| Structured summary | 2 | Provide a structured summary including, as applicable:  **Background:** main objectives  **Methods:** data sources; study eligibility criteria, participants, and interventions; study appraisal; and *synthesis methods, such as network meta-analysis.*  **Results:** number of studies and participants identified; summary estimates with corresponding confidence/credible intervals; treatment rankings may also be discussed. Authors may choose to summarize pairwise comparisons against a chosen treatment included in their analyses for brevity.  **Discussion/Conclusions:** limitations; conclusions and implications of findings.  **Other:** primary source of funding; systematic review registration number with registry name. | 4 |
|  |  |  |  |
| **INTRODUCTION** |  |  |  |
| Rationale | 3 | Describe the rationale for the review in the context of what is already known*, including mention of why a network meta-analysis has been conducted.* | 6 |
| Objectives | 4 | Provide an explicit statement of questions being addressed, with reference to participants, interventions, comparisons, outcomes, and study design (PICOS). | 7 |
|  |  |  |  |
| **METHODS** |  |  |  |
| Protocol and registration | 5 | Indicate whether a review protocol exists and if and where it can be accessed (e.g., Web address); and, if available, provide registration information, including registration number. | NA |
| Eligibility criteria | 6 | Specify study characteristics (e.g., PICOS, length of follow-up) and report characteristics (e.g., years considered, language, publication status) used as criteria for eligibility, giving rationale. *Clearly describe eligible treatments included in the treatment network, and note whether any have been clustered or merged into the same node (with justification).* | 8 |
| Information sources | 7 | Describe all information sources (e.g., databases with dates of coverage, contact with study authors to identify additional studies) in the search and date last searched. | 7 |
| Search | 8 | Present full electronic search strategy for at least one database, including any limits used, such that it could be repeated. | Appendix S3 |
| Study selection | 9 | State the process for selecting studies (i.e., screening, eligibility, included in systematic review, and, if applicable, included in the meta-analysis). | 8, 9 |
| Data collection process | 10 | Describe method of data extraction from reports (e.g., piloted forms, independently, in duplicate) and any processes for obtaining and confirming data from investigators. | 9 |
| Data items | 11 | List and define all variables for which data were sought (e.g., PICOS, funding sources) and any assumptions and simplifications made. | 9 |
| Geometry of the network | S1 | Describe methods used to explore the geometry of the treatment network under study and potential biases related to it. This should include how the evidence base has been graphically summarized for presentation, and what characteristics were compiled and used to describe the evidence base to readers. | 9 |
| Risk of bias within individual studies | 12 | Describe methods used for assessing risk of bias of individual studies (including specification of whether this was done at the study or outcome level), and how this information is to be used in any data synthesis. | 9 |
| Summary measures | 13 | State the principal summary measures (e.g., risk ratio, difference in means). Also describe the use of additional summary measures assessed, such as treatment rankings and surface under the cumulative ranking curve (SUCRA) values, as well as modified approaches used to present summary findings from meta-analyses. | 9 |
| Planned methods of analysis | 14 | Describe the methods of handling data and combining results of studies for each network meta-analysis. This should include, but not be limited to:   - Handling of multi-arm trials; - Selection of variance structure; - Selection of prior distributions in Bayesian analyses; and - Assessment of model fit. | 9 |
| Assessment of Inconsistency | S2 | Describe the statistical methods used to evaluate the agreement of direct and indirect evidence in the treatment network(s) studied. Describe efforts taken to address its presence when found. | 10 |
| Risk of bias across studies | 15 | Specify any assessment of risk of bias that may affect the cumulative evidence (e.g., publication bias, selective reporting within studies). | 9 |
| Additional analyses | 16 | Describe methods of additional analyses if done, indicating which were pre-specified. This may include, but not be limited to, the following:   - Sensitivity or subgroup analyses; - Meta-regression analyses; - Alternative formulations of the treatment network; and - Use of alternative prior distributions for Bayesian analyses (if applicable). | 9 |
|  |  |  |  |
| **RESULTS**† |  |  |  |
| Study selection | 17 | Give numbers of studies screened, assessed for eligibility, and included in the review, with reasons for exclusions at each stage, ideally with a flow diagram. | Appendix S1 |
| Presentation of network structure | S3 | Provide a network graph of the included studies to enable visualization of the geometry of the treatment network. | Figure 1 |
| Summary of network geometry | S4 | Provide a brief overview of characteristics of the treatment network. This may include commentary on the abundance of trials and randomized patients for the different interventions and pairwise comparisons in the network, gaps of evidence in the treatment network, and potential biases reflected by the network structure. | 11-12 |
| Study characteristics | 18 | For each study, present characteristics for which data were extracted (e.g., study size, PICOS, follow-up period) and provide the citations. | Appendix S2 |
| Risk of bias within studies | 19 | Present data on risk of bias of each study and, if available, any outcome level assessment. | Appendix S4 |
| Results of individual studies | 20 | For all outcomes considered (benefits or harms), present, for each study: 1) simple summary data for each intervention group, and 2) effect estimates and confidence intervals. *Modified approaches may be needed to deal with information from larger networks.* | Figures 2-4 Appendix S7 |
| Synthesis of results | 21 | Present results of each meta-analysis done, including confidence/credible intervals. In larger networks, authors may focus on comparisons versus a particular comparator (e.g. placebo or standard care), with full findings presented in an appendix. League tables and forest plots may be considered to summarize pairwise comparisons. If additional summary measures were explored (such as treatment rankings), these should also be presented. | Figures 1-5 |
| Exploration for inconsistency | S5 | Describe results from investigations of inconsistency. This may include such information as measures of model fit to compare consistency and inconsistency models, *P* values from statistical tests, or summary of inconsistency estimates from different parts of the treatment network. | Appendix S6-S8 |
| Risk of bias across studies | 22 | Present results of any assessment of risk of bias across studies for the evidence base being studied. | Appendix S4 |
| Results of additional analyses | 23 | Give results of additional analyses, if done (e.g., sensitivity or subgroup analyses, meta-regression analyses*, alternative network geometries studied, alternative choice of prior distributions for Bayesian analyses,* and so forth). | 12 |
|  |  |  |  |
| **DISCUSSION** |  |  |  |
| Summary of evidence | 24 | Summarize the main findings, including the strength of evidence for each main outcome; consider their relevance to key groups (e.g., healthcare providers, users, and policy-makers). | 13-15 |
| Limitations | 25 | Discuss limitations at study and outcome level (e.g., risk of bias), and at review level (e.g., incomplete retrieval of identified research, reporting bias). *Comment on the validity of the assumptions, such as transitivity and consistency. Comment on any concerns regarding network geometry (e.g., avoidance of certain comparisons).* | 15 |
| Conclusions | 26 | Provide a general interpretation of the results in the context of other evidence, and implications for future research. | 16 |
|  |  |  |  |
| **FUNDING** |  |  | 2 |
| Funding | 27 | Describe sources of funding for the systematic review and other support (e.g., supply of data); role of funders for the systematic review. This should also include information regarding whether funding has been received from manufacturers of treatments in the network and/or whether some of the authors are content experts with professional conflicts of interest that could affect use of treatments in the network. |  |

PICOS = population, intervention, comparators, outcomes, study design.
* Text in italics indicates wording specific to reporting of network meta-analyses that has been added to guidance from the PRISMA statement.
† Authors may wish to plan for use of appendices to present all relevant information in full detail for items in this section.
Table is based on PRISMA guidelines.([28](#_ENREF_28))

**APPENDIX S10. HETEROGENEITY ANALYSIS OF PAIRWISE COMPARSONS WITH PLACEBO**

The table below reports estimates of the I^2^ statistic for heterogeneity for treatment-placebo comparisons with ≥2 independent studies. Confidence intervals are reported for comparisons with ≥3 independent studies.

|  | | ASAS 20 | | | BASFI | | | | CRP | | |  |
| --- | --- | --- | --- | --- | --- | --- | --- | --- | --- | --- | --- | --- |
|  | Number of Studies | | I^2^ | Number of Studies | | I^2^ | | Number of Studies | | | I^2^ | |
| GOL IV 2 mg/kg | 1 | | NA | 1 | | NA | 1 | | | NA | |  |
| ADA 40 mg | 4 | | 56% | 5 | | 0% | 5 | | | 0% | |  |
| CZP 200 mg | 1 | | NA | 1 | | NA | 0 | | | NA | |  |
| CZP 400 mg | 1 | | NA | 1 | | NA | 0 | | | NA | |  |
| ETN 25 mg | 4 | | 0% | 4 | | **90%** | 2 | | | 0% | |  |
| ETN 50 mg | 1 | | NA | 0 | | NA | 0 | | | NA | |  |
| IFX 5 mg/kg | 2 | | 0% | 2 | | 0% | 2 | | | 45% | |  |
| GOL 50 mg | 2 | | 43% | 2 | | 0% | 1 | | | NA | |  |
| GOL 100 mg | 1 | | NA | 1 | | NA | 1 | | | NA | |  |
| SEC SC 75 mg | 1 | | NA | 1 | | NA | 1 | | | NA | |  |
| SEC SC 150 mg | 2 | | 74% | 1 | | NA | 2 | | | 0% | |  |
| SEC IV 75 mg | 1 | | NA | 1 | | NA | 1 | | | NA | |  |
| SEC IV 150 mg | 2 | | 20% | 1 | | NA | 1 | | | NA | |  |
| SEC IV 300 mg | 1 | | NA | 0 | | NA | 0 | | | NA | |  |
| APR 30 mg | 2 | | 55% | 1 | | NA | 0 | | | NA | |  |
| TOF 2 mg | 1 | | NA | 1 | | NA | 1 | | | NA | |  |
| TOF 5 mg | 1 | | NA | 1 | | NA | 1 | | | NA | |  |
| TOF 10 mg | 1 | | NA | 1 | | NA | 1 | | | NA | |  |
| FIL 200 mg | 1 | | NA | 1 | | NA | 1 | | | NA | |  |
| IXE 80 mg Q2W | 2 | | 5% | 2 | | 0% | 2 | | | 76% | |  |
| IXE 80 mg Q4W | 2 | | 0% | 2 | | 0% | 2 | | | 89% | |  |
| RIS 18 mg | 1 | | NA | 0 | | NA | 0 | | | NA | |  |
| RIS 90 mg | 1 | | NA | 0 | | NA | 0 | | | NA | |  |
| RIS 180 mg | 1 | | NA | 0 | | NA | 0 | | | NA | |  |
| UST 45 mg | 2 | | 0% | 2 | | 0% | 2 | | | 0% | |  |
| UST 90 mg | 2 | | 44% | 2 | | 0% | 2 | | | 69% | |  |
| SEC SC 150 mg - no LD | 1 | | NA | 0 | | NA | 1 | | | NA | |  |

Reference List

1. Deodhar A, Reveille JD, Harrison DD, Kim L, Lo KH, Leu JH, et al. Safety and efficacy of golimumab administered intravenously in adults with ankylosing spondylitis: Results through week 28 of the go-alive study. J Rheumatol 2018;45:341-8.

2. Inman RD, Davis JC, Jr., Heijde D, Diekman L, Sieper J, Kim SI, et al. Efficacy and safety of golimumab in patients with ankylosing spondylitis: Results of a randomized, double-blind, placebo-controlled, phase iii trial. Arthritis Rheum 2008;58:3402-12.

3. Bao C, Huang F, Khan MA, Fei K, Wu Z, Han C, et al. Safety and efficacy of golimumab in chinese patients with active ankylosing spondylitis: 1-year results of a multicentre, randomized, double-blind, placebo-controlled phase iii trial. Rheumatology (Oxford) 2014;53:1654-63.

4. Braun J, Brandt J, Listing J, Zink A, Alten R, Golder W, et al. Treatment of active ankylosing spondylitis with infliximab: A randomised controlled multicentre trial. Lancet 2002;359:1187-93.

5. van der Heijde D, Dijkmans B, Geusens P, Sieper J, DeWoody K, Williamson P, et al. Efficacy and safety of infliximab in patients with ankylosing spondylitis: Results of a randomized, placebo-controlled trial (assert). Arthritis Rheum 2005;52:582-91.

6. van der Heijde D, Kivitz A, Schiff MH, Sieper J, Dijkmans BA, Braun J, et al. Efficacy and safety of adalimumab in patients with ankylosing spondylitis: Results of a multicenter, randomized, double-blind, placebo-controlled trial. Arthritis Rheum 2006;54:2136-46.

7. Maksymowych WP, Rahman P, Shojania K, Olszynski WP, Thomson GT, Ballal S, et al. Beneficial effects of adalimumab on biomarkers reflecting structural damage in patients with ankylosing spondylitis. J Rheumatol 2008;35:2030-7.

8. Hu Z, Xu M, Li Q, Lin Z, Liao Z, Cao S, et al. Adalimumab significantly reduces inflammation and serum dkk-1 level but increases fatty deposition in lumbar spine in active ankylosing spondylitis. Int J Rheum Dis 2012;15:358-65.

9. Huang F, Gu J, Zhu P, Bao C, Xu J, Xu H, et al. Efficacy and safety of adalimumab in chinese adults with active ankylosing spondylitis: Results of a randomised, controlled trial. Ann Rheum Dis 2014;73:587-94.

10. Landewe R, Braun J, Deodhar A, Dougados M, Maksymowych WP, Mease PJ, et al. Efficacy of certolizumab pegol on signs and symptoms of axial spondyloarthritis including ankylosing spondylitis: 24-week results of a double-blind randomised placebo-controlled phase 3 study. Ann Rheum Dis 2014;73:39-47.

11. Gorman JD, Sack KE, Davis JC, Jr. Treatment of ankylosing spondylitis by inhibition of tumor necrosis factor alpha. N Engl J Med 2002;346:1349-56.

12. Davis JC, Jr., Van Der Heijde D, Braun J, Dougados M, Cush J, Clegg DO, et al. Recombinant human tumor necrosis factor receptor (etanercept) for treating ankylosing spondylitis: A randomized, controlled trial. Arthritis Rheum 2003;48:3230-6.

13. Calin A, Dijkmans BA, Emery P, Hakala M, Kalden J, Leirisalo-Repo M, et al. Outcomes of a multicentre randomised clinical trial of etanercept to treat ankylosing spondylitis. Ann Rheum Dis 2004;63:1594-600.

14. van der Heijde D, Da Silva JC, Dougados M, Geher P, van der Horst-Bruinsma I, Juanola X, et al. Etanercept 50 mg once weekly is as effective as 25 mg twice weekly in patients with ankylosing spondylitis. Ann Rheum Dis 2006;65:1572-7.

15. Barkham N, Coates LC, Keen H, Hensor E, Fraser A, Redmond A, et al. Double-blind placebo-controlled trial of etanercept in the prevention of work disability in ankylosing spondylitis. Ann Rheum Dis 2010;69:1926-8.

16. Deodhar A, Gensler LS, Sieper J, Clark M, Calderon C, Wang Y, et al. Three multicenter, randomized, double-blind, placebo-controlled studies evaluating the efficacy and safety of ustekinumab in axial spondyloarthritis. Arthritis rheumatol 2018;2018 Sep 18.

17. Baeten D, Ostergaard M, Wei JC, Sieper J, Jarvinen P, Tam LS, et al. Risankizumab, an il-23 inhibitor, for ankylosing spondylitis: Results of a randomised, double-blind, placebo-controlled, proof-of-concept, dose-finding phase 2 study. Annals of the Rheumatic Diseases 2018;77:1295-302.

18. van der Heijde D, Cheng-Chung WJ, Dougados M, Mease P, Deodhar A, Maksymowych WP, et al. Ixekizumab, an interleukin-17a antagonist in the treatment of ankylosing spondylitis or radiographic axial spondyloarthritis in patients previously untreated with biological disease-modifying anti-rheumatic drugs (coast-v): 16 week results of a phase 3 randomised, double-blind, active-controlled and placebo-controlled trial. Lancet 2018;2018 Oct 22.

19. Deodhar A, Poddubnyy D, Pacheco-Tena C, Salvarani C, Lespessailles E, Rahman P, et al. Efficacy and safety of ixekizumab in the treatment of radiographic axial spondyloarthritis: 16 week results of a phase 3 randomized, double-blind, placebo controlled trial in patients with prior inadequate response or intolerance to tumor necrosis factor inhibitors. Arthritis rheumatol 2018;2018 Oct 20.

20. Deodhar AA, Dougados M, Baeten DL, Cheng-Chung Wei J, Geusens P, Readie A, et al. Effect of secukinumab on patient-reported outcomes in patients with active ankylosing spondylitis: A phase iii randomized trial (measure 1). Arthritis Rheumatol 2016;68:2901-10.

21. Marzo-Ortega H, Sieper J, Kivitz A, Blanco R, Cohen M, Martin R, et al. Secukinumab and sustained improvement in signs and symptoms of patients with active ankylosing spondylitis through two years: Results from a phase iii study. Arthritis Care Res (Hoboken) 2017;69:1020-9.

22. Pavelka K, Kivitz A, Dokoupilova E, Blanco R, Maradiaga M, Tahir H, et al. Efficacy, safety, and tolerability of secukinumab in patients with active ankylosing spondylitis: A randomized, double-blind phase 3 study, measure 3. Arthritis Res Ther 2017;19:285.

23. Kivitz AJ, Wagner U, Dokoupilova E, Supronik J, Martin R, Talloczy Z, et al. Efficacy and safety of secukinumab 150 mg with and without loading regimen in ankylosing spondylitis: 104-week results from measure 4 study. RheumatolTher 2018;Aug 18.

24. van der Heijde D, Baraliakos X, Gensler LS, Maksymowych WP, Tseluyko V, Nadashkevich O, et al. Efficacy and safety of filgotinib, a selective janus kinase 1 inhibitor, in patients with active ankylosing spondylitis (tortuga): Results from a randomised, placebo-controlled, phase 2 trial. Lancet 2018;2018 Oct 22.

25. van der Heijde D, Deodhar A, Wei JC, Drescher E, Fleishaker D, Hendrikx T, et al. Tofacitinib in patients with ankylosing spondylitis: A phase ii, 16-week, randomised, placebo-controlled, dose-ranging study. Ann Rheum Dis 2017;76:1340-7.

26. Pathan E, Abraham S, Van Rossen E, Withrington R, Keat A, Charles PJ, et al. Efficacy and safety of apremilast, an oral phosphodiesterase 4 inhibitor, in ankylosing spondylitis. Ann Rheum Dis 2013;72:1475-80.

27. Study of apremilast to treat subjects with active ankylosing spondylitis. <Https://clinicaltrialsgov/show/nct01583374> 2012.

28. Hutton B SG, Caldwell DM, Chaimani A, Schmid CH, Cameron C, Ioannidis JP, Straus S, Thorlund K, Jansen JP, Mulrow C, Catalá-López F, Gøtzsche PC, Dickersin K, Boutron I, Altman DG, Moher D. The prisma extension statement for reporting of systematic reviews incorporating network meta-analyses of health care interventions: Checklist and explanations. Annals of Internal Medicine 2015;162:777-84.
